# Terrestrial Locomotion in Extant Marine Tetrapods with Flippers

**DOI:** 10.3390/biology15201890

**Published:** 2026-10-20

**Authors:** Vincent Bels, Glenn Le Floch, Florence Kirchhoff, Maëlle Rivain, Laura Leroy, Nikos Vallianos, Chanel Comis, Eric Pelle, Julien Pfyffer, Philippa Agnew, John Davenport

**Affiliations:** 1 Muséum national d’Histoire naturelle, Institut de Systématique Evolution Biodiversité (ISYEB), UMR 7205 MNHN/CNRS/UPMC/EPHE/UA, Sorbonne Université, 75005 Paris, France; glenn.lefloch@mnhn.fr (G.L.F.); maellerivain@sfr.fr (M.R.);; 2 Wildlife Sense, Minies, 28100 Argostoli, Kefalonia, Greece; 3 Direction de Collections, Muséum national d’Histoire naturelle, 75005 Paris, France; 4 Octopus Foundation, 1007 Lausanne, Switzerland; 5 Ōamaru Penguins, Ōamaru 9400, New Zealand; 6 School of Biological, Earth and Environmental Sciences, University College Cork, T23 TK30 Cork, Ireland; j.davenport@ucc.ie

**Keywords:** evolution, flippers, marine tetrapods, terrestrial locomotion, gait, phylogeny

## Abstract

The repeated evolutionary transitions of tetrapods from terrestrial to marine environments represent one of the most remarkable examples of functional convergence among vertebrates. Despite profound specialisation for aquatic locomotion, many marine tetrapods must periodically return to land for essential activities such as reproduction, moulting, and resting, providing a unique opportunity to investigate whether convergent adaptations for swimming have been accompanied by convergent evolution of terrestrial locomotor repertoires. This study combines a comparative synthesis with original phylogenetic analyses to investigate the evolution of terrestrial locomotor repertoires in the three principal lineages of extant marine tetrapods with flippers: sea turtles, penguins, and pinnipeds. A standardised behavioural dataset compiled from published studies, publicly available videos, and original observations was used to reconstruct the evolutionary history of locomotor behaviours, test correlated evolution among locomotor modes, and evaluate the influence of body mass within a phylogenetic framework. Although these lineages exhibit remarkable convergence in appendicular adaptations for aquatic propulsion, our comparative analyses demonstrate that this convergence has not been accompanied by convergence in terrestrial locomotor repertoires. Instead, each lineage has retained or evolved distinct terrestrial locomotor strategies reflecting different combinations of phylogenetic inheritance, biomechanical constraints, and ecological specialisation. Marine turtles exhibit the most conservative terrestrial repertoire, penguins combine highly specialised underwater flight with diverse terrestrial behaviours, and pinnipeds display the greatest diversity of terrestrial locomotor strategies. These findings indicate that similar functional demands for efficient swimming have generated multiple evolutionary solutions for terrestrial locomotion rather than a single convergent strategy. More broadly, this study provides an integrative framework for understanding how behaviour, biomechanics, ecology, and evolutionary history interact to shape locomotor evolution in secondarily aquatic tetrapods and establishes a behavioural foundation for future studies linking structure, performance, behaviour, and fitness.

## Simple Summary

Marine turtles, penguins, and pinnipeds independently evolved flippers that provide efficient aquatic propulsion, collectively offering one of the clearest examples of convergent evolution among vertebrates. However, all must still return to land to reproduce, rest, or moult. This review investigates whether convergent adaptations for swimming have also led to convergent terrestrial locomotor repertoires. By combining published studies, original observations, and phylogenetic analyses, we show that species within and among these three lineages have evolved distinct terrestrial locomotor repertoires shaped by their evolutionary history, biomechanical constraints, and ecology. These findings indicate that similar adaptations for swimming do not necessarily produce similar ways of moving on land.

## 1. Introduction

### 1.1. Overview of Extant Marine Tetrapods

**Figure 1:**
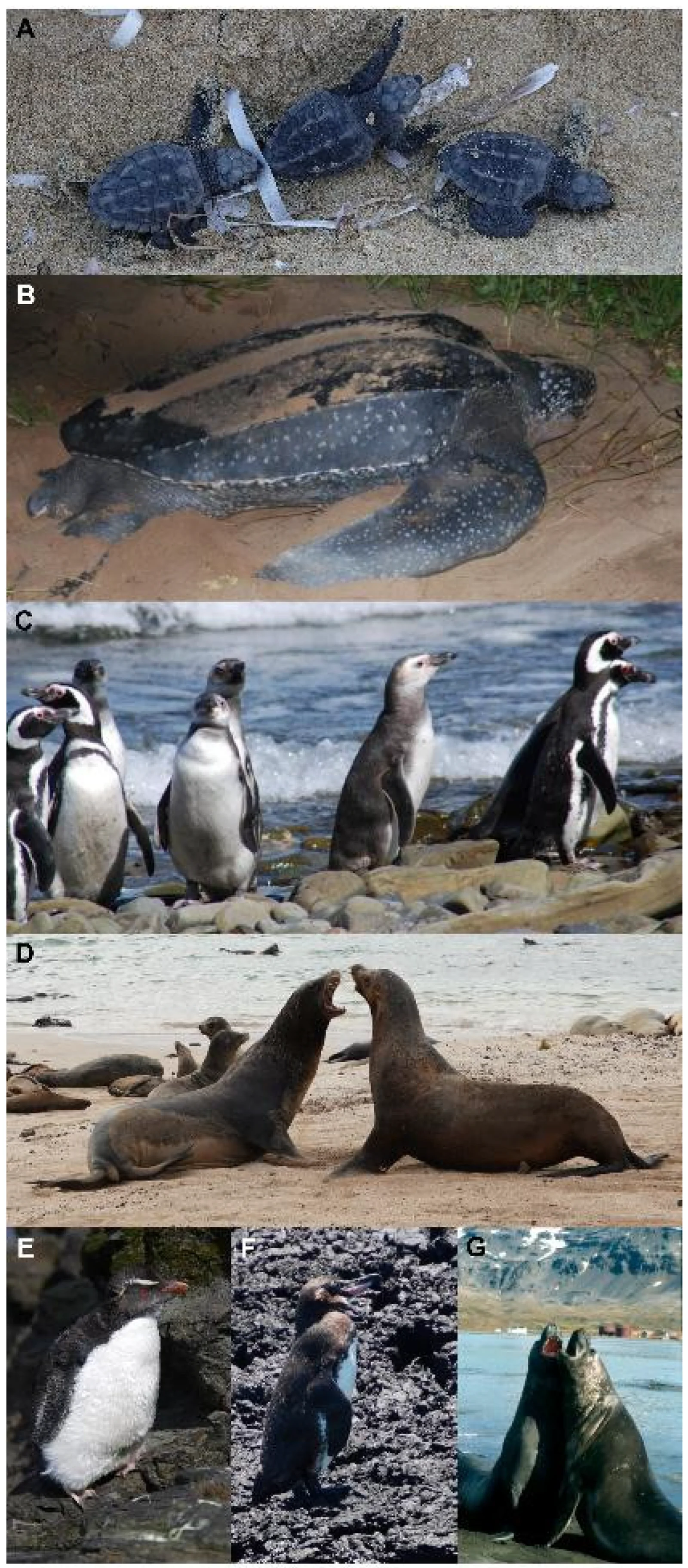
Representative extant marine tetrapods with flippers that have convergently evolved specialised locomotor systems for aquatic life while regularly returning to land. (**A**) Hatchling *Caretta caretta* on Kefalonia Island, Greece. Photo credit: Florence Kirchhoff. (**B**) Adult female *Dermochelys coriacea* in French Guiana. Photo credit: Eric Pellé. (**C**) *Spheniscus humboldti* in Patagonia. Photo credit: Eric Pellé. (**D**) *Eudyptes chrysocome* in Patagonia. Photo credit: Eric Pellé. (**E**). *Mirounga leonina* on South Georgia Island. Photo credit: Eric Pellé. (**D**) *Zalophus wollebaeki* on the beach of Santa Cruz (Galapagos island). Photo credit: Florence Kirchhoff (**E**) *Eudyptes chrysocome*. Photo credit: Florence Kirchhoff. (**F**) *Spheniscus mendiculus* on Isabella (Galapagos island). Photo credit: Florence Kirchhoff. (**G**) *Mirounga leonina* on South Georgia Island. Photo credit: John Davenport.

Throughout tetrapod evolution, numerous extinct and extant lineages independently returned from terrestrial to aquatic environments after evolving from four-limbed terrestrial ancestors (Figure 1). These independent transitions gave rise to a broad diversity of life histories, ranging from fully aquatic taxa, such as extinct ichthyosaurs and extant cetaceans and sirenians, to lineages that continue to depend on both aquatic and terrestrial environments, including the three extant lineages of marine tetrapods with flippers considered in this study: sea turtles, penguins, and pinnipeds [1,2,3,4,5,6,7,8,9,10]. Despite their independent evolutionary origins, these groups exhibit remarkable morphological, physiological, and biomechanical adaptations that enable efficient locomotion in water while retaining, to varying degrees, the ability to move on land.

**Figure 2:**
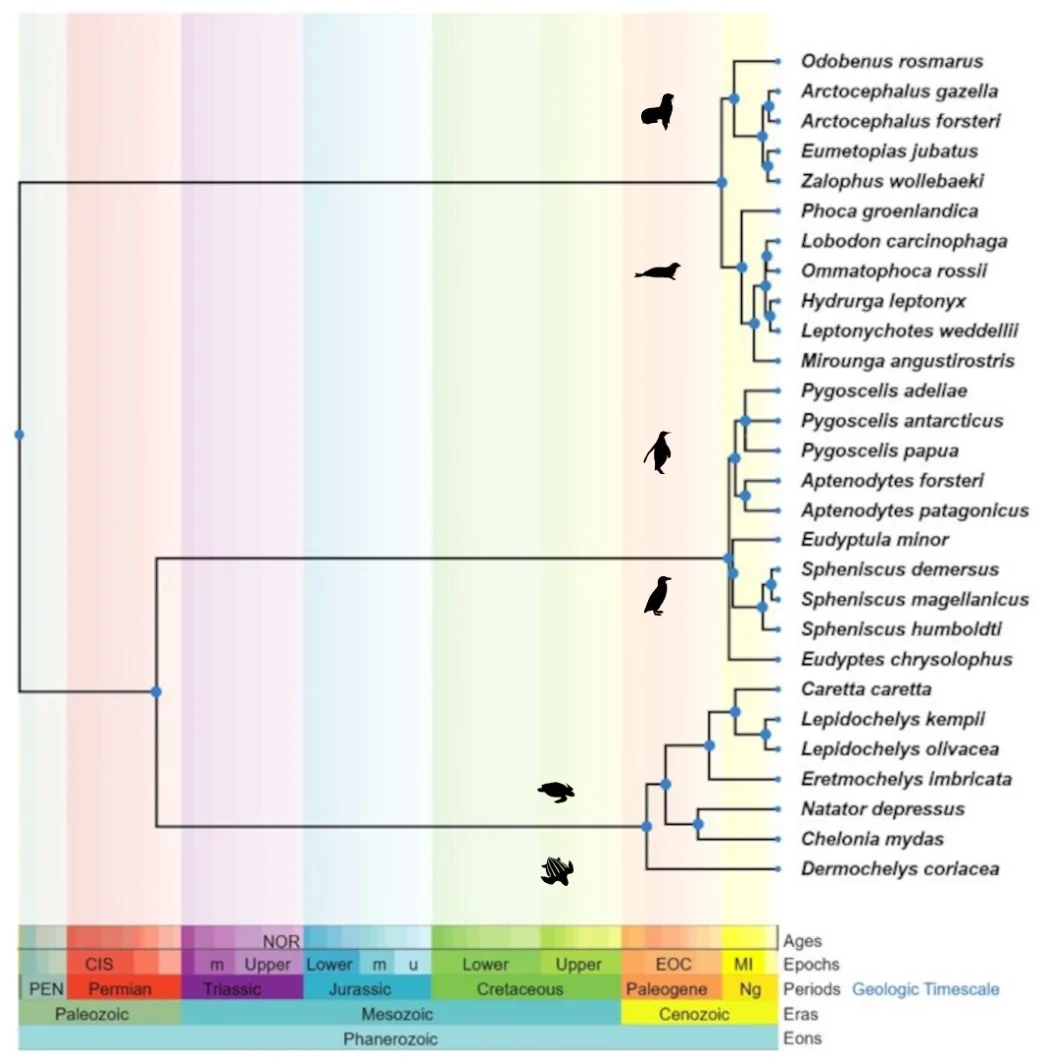
Time-calibrated phylogeny of extant marine tetrapods with flippers illustrating the independent evolutionary transitions from terrestrial to marine environments in sea turtles, penguins, and pinnipeds. All lineages retain regular terrestrial locomotion for reproduction, as well as for moulting in penguins and pinnipeds. Silhouettes of all species are from PhyloPic (https://www.phylopic.org/ [Phylopics for pinnipeds: https://www.phylopic.org/images/d78cfdec-118d-475e-a97f-185a82df18c0/arctocephalus-tropicalis; https://www.phylopic.org/images/41d9f21e-7e0b-42d2-a088-e154bfa63984/phoca-largha; for penguins: https://www.phylopic.org/nodes/ddfea46d-cc05-4420-90a6-95a5c411e7d0/spheniscus-silhouettes; https://www.phylopic.org/images/4c20cc68-1acd-4c5a-adfe-86a61609748b/aptenodytes-patagonicus; for marine turtles: https://www.phylopic.org/images/146add90-a7f4-4735-8c31-90e6604cc8f4/lepidochelys-kempii; https://www.phylopic.org/images/6972a938-f77b-4ec6-a1d3-8fe49942f7b3/dermochelys-coriacea]).

Marine turtles (Cheloniidae and Dermochelyidae), penguins (Spheniscidae), and pinnipeds (Phocidae, Otariidae, and Odobenidae) provide some of the clearest examples of convergent evolution among vertebrates. Although their terrestrial ancestors differed markedly in anatomy and locomotor patterns, all three lineages independently evolved forelimbs specialised as flippers for aquatic propulsion. This convergence reflects the strong selective pressures imposed by locomotion in water, resulting in similar functional solutions despite distinct phylogenetic origins [4,7,8,11,12,13]. At the same time, important differences remain in the organisation and contribution of the fore- and hindlimbs, producing distinct swimming modes among the three groups (Figure 2).

These convergent transformations involve profound modifications to the musculoskeletal system. In marine turtles, elongated and stiffened foreflippers generate lift-based propulsion during both downstroke and upstroke, with asymmetric force production optimising thrust while minimising drag [14,15,16,17]. Penguins have transformed their wings into hydrodynamic flippers for underwater flight through modification of their skeletal architecture, increased bone density, and extensive muscular specialisation [18,19,20,21,22,23]. Pinnipeds display greater functional diversity: otariids primarily use their foreflippers for propulsion, whereas phocids rely mainly on hindlimb-driven swimming, with the forelimbs contributing principally to stabilisation [24,25,26,27,28,29,30]. Together, these examples illustrate how convergent adaptation to aquatic life has produced different biomechanical solutions while preserving lineage-specific locomotor characteristics.

Despite their specialisation for aquatic locomotion, extant marine tetrapods with flippers must regularly return to land. Sea turtles emerge exclusively for nesting, whereas penguins and pinnipeds also use terrestrial habitats for breeding, moulting, resting, and, in some species, predator avoidance. Consequently, terrestrial locomotion remains essential in their life history, although the environmental conditions encountered on land can differ among taxa, ranging from sandy beaches to rocky shores, snow, and ice. Terrestrial locomotion is therefore constrained both by the ancestral locomotor organisation inherited from terrestrial ancestors and by the ecological characteristics of the habitats currently occupied. Moreover, increasing coastal modification resulting from climate change and human activities is altering many of these habitats, and may directly affect terrestrial movements associated with reproduction and survival [31,32,33]. Despite the fundamental importance of terrestrial locomotion for completing the life cycle of these marine vertebrates, its evolutionary diversification has received considerably less attention than aquatic locomotion.

### 1.2. Constraints on Locomotion in Aquatic and Terrestrial Habitats

The evolutionary transition from terrestrial to aquatic locomotion requires profound modifications of ancestral tetrapod locomotor systems. It has been suggested that the earliest swimming behaviours were derived from terrestrial gaits through progressive modifications of rhythmic limb movements. Limb-driven paraxial paddling, for example, is considered a transformation of terrestrial locomotion in which rhythmic fore- and hindlimb movements generate aquatic propulsion [2,5,7,34]. Quadrupedal paddling (“dog paddle”) is generally viewed as a modified terrestrial diagonal gait, whereas bipedal paddling represents a further specialisation in which forelimb movements alone generate propulsion, thereby reducing interference between ipsilateral limbs. Interestingly, polar bears, which represent a close terrestrial relative of pinnipeds, also swim using predominantly alternate forelimb paddling, illustrating how aquatic propulsion may evolve from terrestrial locomotor patterns without complete morphological specialisation.

The return to land by secondarily aquatic tetrapods presents an equally demanding biomechanical challenge. In contrast to aquatic locomotion, terrestrial movement requires the locomotor system to support body weight against gravity while simultaneously generating propulsion through frictional interactions with the substratum [35]. Depending on the species and habitat, terrestrial locomotion involves either discrete contacts between the distal limbs and the substratum or continuous support of the body by its ventral surface. Consequently, marine tetrapods moving on land must perform terrestrial locomotion using appendicular systems that have undergone profound evolutionary specialisation for swimming.

These contrasting mechanical environments have driven convergent transformations of skeletal morphology, musculoskeletal organisation, and locomotor function throughout the evolution of secondarily aquatic tetrapods [1,4,7,23,36,37]. Understanding how terrestrial locomotor repertoires have been maintained or modified despite these profound morphological transformations requires an integrative approach combining comparative biomechanics, behaviour, and phylogenetic analyses [4,5,38,39,40]. This conceptual framework forms the basis of the comparative analyses presented in this study.

### 1.3. Objectives

The present study aims to determine whether convergent adaptations for aquatic propulsion have led to common or lineage-specific terrestrial locomotor repertoires. Specifically, we compare the terrestrial locomotor behaviours of extant marine turtles, penguins, and pinnipeds, reconstruct the evolution of individual locomotor modes, test for evolutionary associations among locomotor behaviours, and assess the influence of body mass on the evolution of terrestrial locomotor repertoires. We evaluate two alternative hypotheses: (1) convergent specialisation for aquatic propulsion was accompanied by convergence in terrestrial locomotor repertoires; or (2) terrestrial locomotor repertoires remained lineage-specific despite convergence in aquatic propulsion.

To address these objectives, we first provide a comprehensive synthesis of current knowledge on terrestrial locomotion by integrating published studies, publicly available video observations, and original observations. We then use a standardised behavioural dataset to perform phylogenetic comparative analyses, including ancestral-state reconstructions, tests of correlated evolution among locomotor behaviours, and assessments of body-mass effects. Together, these complementary approaches provide an integrative framework for evaluating whether and how adaptation to aquatic life has influenced the evolution of terrestrial locomotion in secondarily aquatic tetrapods.

## 2. Methods

### 2.1. Literature and Video Survey

The present study combined a comprehensive survey of the published literature, original field observations, and analyses of publicly available video material to compile a comparative dataset of terrestrial locomotor behaviours in extant marine tetrapods with flippers. The literature and video survey provided the biological and biomechanical synthesis, whereas the resulting species-level behavioural dataset was used in the original comparative and phylogenetic analyses. The objective was to assemble the most comprehensive behavioural dataset currently available for the three lineages considered in this study.

The literature survey was conducted iteratively using Google Scholar and Web of Science, and was last updated on 1 August 2026. Searches were performed separately for marine turtles, penguins, and pinnipeds. Taxonomic terms, including Cheloniidae, Dermochelyidae, Spheniscidae, Phocidae, Otariidae, Odobenidae, and individual species names, were combined with locomotion-related terms, including “terrestrial locomotion”, “quadrupedal locomotion”, “bipedal locomotion”, “synchronous locomotion”, “asynchronous locomotion”, “walking”, “gait”, “jumping”, “hopping”, “tobogganing”, “biomechanics”, “kinematics”, and “locomotor behaviour”. Searches were not restricted by publication date. Additional publications were identified by examining the reference lists of relevant reviews and monographs using an iterative snowball procedure. Publications were included in the biological synthesis when they provided information on terrestrial or aquatic locomotion, biomechanics, functional morphology, or ecology relevant to the objectives of the study. More restrictive criteria were applied when constructing the behavioural dataset used in the comparative analyses: a publication was used to establish a character state only when it reported an original observation or an unambiguous description of the locomotor behaviour concerned. Anecdotal statements that did not allow the behaviour to be identified reliably were not used for character coding. Review articles, monographs, and book chapters were consulted for general syntheses, historical information, and references to primary studies that were not readily accessible.

Body-mass values were compiled exclusively from peer-reviewed publications and authoritative species-specific references. The principal sources used to compile body-mass values are provided in Table 1, Table 2 and Table 3. Behavioural observations were obtained primarily from publicly available online videos (predominantly from YouTube, but also from the BBC, National Geographic, and other documentary or commercial sources; see Supplementary Material File S1A–C). Videos were retained only when complete locomotor cycles could be clearly observed and analysed. Whenever possible, video observations were cross-checked against published descriptions to ensure consistency. Video sequences were downloaded using ClipGrab (version 3.0.15; https://clipgrab.org) and analysed frame by frame with Kinovea (version 2025.2.0; https://www.kinovea.org), allowing detailed examination of limb movements, body posture, and locomotor sequences. The dataset was supplemented by original observations of free-ranging animals in French Guiana, Patagonia, and New Zealand, and of captive individuals maintained at the Parc Zoologique de Branféré (Le Guerno, France) and Océanopolis (Brest, France). Animals were filmed at 60–120 frames s^−1^ using Sony DSC-RX10 IV (Sony Corporation, Tokyo, Japan) and Nikon Z9 cameras (Nikon Corporation, Tokyo, Japan). The Nikon Z9 was equipped with a Tamron 150–600 mm lens (Tamron Co., Ltd., Saitama, Japan). The resulting sequences were examined frame by frame using Kinovea (https://www.kinovea.org) to identify limb movements, body posture, and locomotor patterns in available species.

**Table 3:** Comparison of body masses and modes of terrestrial locomotion in adult pinnipeds (Phocidae, Otariidae, and Odobenidae). P indicates that a locomotor mode was present in a species; A indicates that it was absent. Adult body masses correspond to median values calculated from published body-mass ranges.

Species	Common Name	Adult Mass Range (kg)	Adult Median Mass (kg)	Movement Without Limb Use	Synchronous Forelimb	Asynchronous Forelimb	Synchronous Hindlimb	Asynchronous Hindlimb	Bipedal	Quadrupedal	Ventral Surface	Walk	Roll	Dorsoventral Undulation	Lateral Undulation
Phocidae															
*Mirounga angustirostris*	Northern elephant seal	800–3000	1900	A	P	A	A	A	P	A	P	A	A	P	A
*Mirounga leonina*	Southern elephant seal *	900–4000	2450	P	P	A	A	A	P	A	P	A	A	P	A
*Leptonychotes weddellii*	Weddell seal	400–600	500	P	P	A	A	A	P	A	P	A	A	P	P
*Hydrurga leptonyx*	Leopard seal	200–600	400	P	P	P	A	A	P	P	P	A	A	P	P
*Ommatophoca rossii*	Ross seal	150–215	183	P	P	A	A	A	P	A	P	A	A	P	A
*Lobodon carcinophaga*	Crabeater seal	200–215	208	P	P	P	A	A	P	P	P	A	A	P	P
*Erignathus barbatus*	Bearded seal	200–400	300	A	P	P	A	A	P	A	P	A	A	P	A
*Cystophora cristata*	Hooded seal	300–410	355	P	P	A	A	A	P	A	P	A	A	P	A
*Pagophilus groenlandicus*	Harp seal **	115–140	140	A	P	P	A	A	P	A	P	A	A	P	P
*Histiophoca fasciata*	Ribbon seal	70–110	110	P	P	A	A	A	P	A	P	A	P	P	A
*Phoca largha*	Spotted seal	82–109	88	P	P	A	A	A	P	A	P	A	A	P	A
*Pusa hispida*	Ringed seal	50–70	60	P	P	P	A	A	P	A	P	A	A	P	A
Otariidae															
*Zalophus wollebaeki*	Galápagos sea lion	50–240	145	A	P	P	P	P	A	P	A	P	A	P	P
*Eumetopias jubatus*	Steller sea lion	240–1120	680	A	P	P	P	P	A	P	A	P	A	P	P
*Arctocephalus forsteri*	New Zealand fur seal	30–160	95	A	P	P	P	P	A	P	A	P	A	P	P
*Arctocephalus gazella*	Antarctic fur seal	34–133	83.5	A	P	P	P	P	A	P	A	P	A	P	P
Odobenidae															
*Odobenus rosmarus*	Walrus ***	560–2300	1430	A	A	P	A	P	P	P	P	A	P	P	P
Possible outgroups															
*Ursus maritimus*	Polar bear ****	300–800	-	A	P	P	P	P	P	P	P	P	P	P	P
*Gulo gulo*	Wolverine *****	9–25	-	A	P	P	P	P	A	P	P	P	P	P	P

* Very large bull southern elephant seals sometimes move down beaches in South Georgia without their foreflippers touching the substratum (JD, pers. obs.). ** Harp seals show side-to-side movements of the body, which have been described as lateral undulations [54], but may reflect alternate action of the forelimbs, rather than being propulsive (see text). *** Adult walruses have recently been seen to roll deliberately down sandy beaches, a novel form of rapid locomotion (BBC 2022—Table S1). **** [55]; ***** BBC (2006). https://www.youtube.com/watch?v=b5Dlxn_tRyM&t=6s. URL last accessed on 7 October 2026 Body-mass data were compiled from authoritative taxonomic monographs and species accounts [29,56,57,58,59]. Because the aim of this study was to assemble a comprehensive comparative behavioural dataset rather than conduct a formal systematic review or meta-analysis, the literature survey was designed to maximise coverage of the available evidence rather than to follow a PRISMA workflow. For the phylogenetic analyses, each locomotor behaviour was coded as a discrete binary character (presence/absence; Table 1, Table 2 and Table 3). A behaviour was scored as present only when it could be unambiguously identified from published descriptions, original observations, or complete locomotor cycles extracted from video sequences. Ambiguous, anecdotal, or isolated observations were excluded. Binary coding was adopted because the available information originated from heterogeneous sources with widely differing levels of quantitative detail, precluding standardised quantitative comparisons across all species. Similar comparative approaches combining published information with publicly available photographic and video resources have recently been applied successfully to investigate the evolution of behavioural traits across broad taxonomic datasets [60]. Operational definitions of all locomotor characters used for behavioural coding are provided in Table 4.

**Table 4:** Definitions of locomotor characters used for behavioural coding in the phylogenetic comparative analyses and their occurrence in extant marine turtles, penguins, and pinnipeds. ✓ indicates presence of behavioural character.

Behavioural Character	Definition	Marine Turtles	Penguins	Pinnipeds
Quadrupedal locomotion	Locomotion involving coordinated support and propulsion by both fore- and hindlimbs.	–	–	✓
Bipedal locomotion	Locomotion in which propulsion is generated by a single limb pair: the hindlimbs in penguins or the forelimbs in phocids; other body regions may also contribute to support.	–	✓	✓
Asynchronous forelimb locomotion	Propulsion produced by alternating actions of the left and right forelimbs.	✓	–	✓
Synchronous forelimb locomotion	Propulsion produced by simultaneous actions of both forelimbs.	✓	–	–
Walking	Progression produced by successive alternating limb support phases.	–	✓	✓
Jumping	Simultaneous take-off of both hindlimbs followed by a distinct aerial phase before landing.	–	✓	–
Hopping	Series of successive jumps performed without an intermediate preparatory phase between consecutive take-offs.	–	✓	–
Tobogganing	Ventral sliding of the body over the substratum assisted by action of the limbs.	–	✓	✓

### 2.2. Analyses

#### 2.2.1. Phylogenetic Comparative Methods

Phylogenetic tree preparation and comparative analyses were conducted in R following the procedures described by Revell and Harmon [61], using the packages *ape*, *geiger*, *phytools*, and *ggplot2* [62,63,64,65]. Separate phylogenetic trees were prepared for marine turtles, penguins, and pinnipeds. The marine-turtle and pinniped trees were obtained from TimeTree [66] and contained, respectively, the seven and 17 extant species represented in the behavioural dataset. The penguin phylogeny was obtained from Cole et al. [67] and pruned to retain the 17 species represented in our dataset. Species names in each behavioural dataset were checked against the corresponding phylogenetic tree before analysis. Figures presenting phylogenetic reconstructions and body-mass analyses were generated in R (version 4.6.0) using functions from *phytools* and *ggplot2*, and were subsequently imported into Inkscape and GIMP for graphical adjustments.

#### 2.2.2. Ancestral-State Reconstruction

From the phylogenetic trees of each taxon, the evolution of a single locomotor feature (as presence/absence) was modelled using the *fitMk* function from the *phytools* package [62], which implements the Mk model for discrete character evolution [68]. Four models were tested: (1) the Equal-Rates model (ER), in which all transition rates between states are assumed to be equal; (2) the All-Rates-Different model (ARD), in which each transition has its own rate; and two constrained models allowing transitions in only one direction (either from state *a* to *b*, or from *b* to *a*). Model selection was primarily based on the corrected Akaike information criterion (AICc), which accounts for potential overfitting due to the small sample size in each taxon. To ensure robustness of model choice, the Bayesian information criterion (BIC) was also computed and compared. For each selected model, 1000 stochastic character maps were simulated on the corresponding phylogenetic tree. One map out of every ten simulations was retained, yielding a subset of 100 maps used to estimate ancestral-state probabilities and visualise state changes along branches [69]. Based on those trees, the ancestral state at each node was also estimated with posterior probabilities [61].

#### 2.2.3. Multiple Character Evolution

Penguins and pinnipeds exhibit several distinct locomotor behaviours, allowing evolutionary associations among characters to be examined within each lineage. Pairwise tests of correlated evolution were performed using Pagel’s discrete-character model [70], implemented with the *fitPagel* function in the R package *phytools*. For each pair of binary characters, a dependent model, in which the transition rates of one character were allowed to vary according to the state of the other character, was compared with an independent model, in which the two characters evolved independently. Model fit was compared using Akaike information criterion (AIC) values, and statistical support for the dependent model was evaluated using the likelihood-ratio test implemented by *fitPagel*. Because multiple character pairs were examined in datasets containing relatively few species, statistically significant or near-significant associations were interpreted cautiously as comparative evidence of correlated evolution rather than as demonstrations of causal relationships between behaviours.

#### 2.2.4. Mass Factor in Locomotion

For each of the three lineages, the effect of phylogeny on body mass was tested using Blomberg’s K statistic [71]. The relationship between body mass and the presence or absence of individual locomotor characters was then examined using phylogenetic logistic regression, implemented with the *phyloglm* function. In these models, the presence or absence of the locomotor character was the binary response variable, and body mass was the explanatory variable, with phylogenetic relationships among species taken into account. For comparison, differences in body mass between species exhibiting or lacking each locomotor character were also evaluated using non-phylogenetic Mann–Whitney U tests. Analyses were not performed when one of the two character one of the two-character states was represented by too few species to permit a meaningful statistical comparison. The significance threshold was set at α = 0.05.

#### 2.2.5. Scope and Interpretation of the Comparative Analyses

The species numbers and character-state distributions used in the comparative analyses reflect the extant biological diversity of each lineage and the documented locomotor repertoire of each species. Each species contributed a single presence or absence state for each locomotor character, irrespective of the number of publications, videos, or original observations documenting that state. Differences in the number of species exhibiting particular behaviours are therefore biological properties of the lineages rather than consequences of an experimental sampling design. Some locomotor characters are restricted to a small number of taxa, whereas others are widespread or entirely absent from particular lineages because of their morphology, locomotor organisation, and evolutionary history.

These biologically determined distributions affect the statistical resolution of comparative analyses. When one character state occurs in only a few extant species, estimates of transition rates, ancestral-states, or evolutionary associations are necessarily less precise. Consequently, the analyses presented here describe phylogenetic patterns in the distribution of documented locomotor behaviours and provide evidence for evolutionary hypotheses, but they should not be interpreted as direct demonstrations of causal evolutionary mechanisms.

## 3. Results

### 3.1. Turtles

Sea turtles are the largest extant fully marine reptiles. They have much broader geographical ranges than most of their freshwater and terrestrial relatives, and are capable of rapid, long-distance aquatic locomotion [72]. Seven extant species are recognised in two families: *Dermochelys coriacea*, the sole extant representative of Dermochelyidae, and *Eretmochelys imbricata*, *Lepidochelys kempii*, *L. olivacea*, *Caretta caretta*, *Chelonia mydas*, and *Natator depressus* in Cheloniidae. These lineages descend from chelonian ancestors capable of both terrestrial and aquatic locomotion [73]. Phylogenetic relationships within Cheloniidae remain partly unresolved [74,75,76], with different molecular datasets supporting alternative hypotheses. For example, *Natator depressus* has been recovered either as the sister taxon to a clade comprising *Eretmochelys*, *Caretta*, and *Lepidochelys*, or as the sister taxon to *Chelonia*.

#### 3.1.1. Emerging from the Egg and the Nest

**Figure 3:**
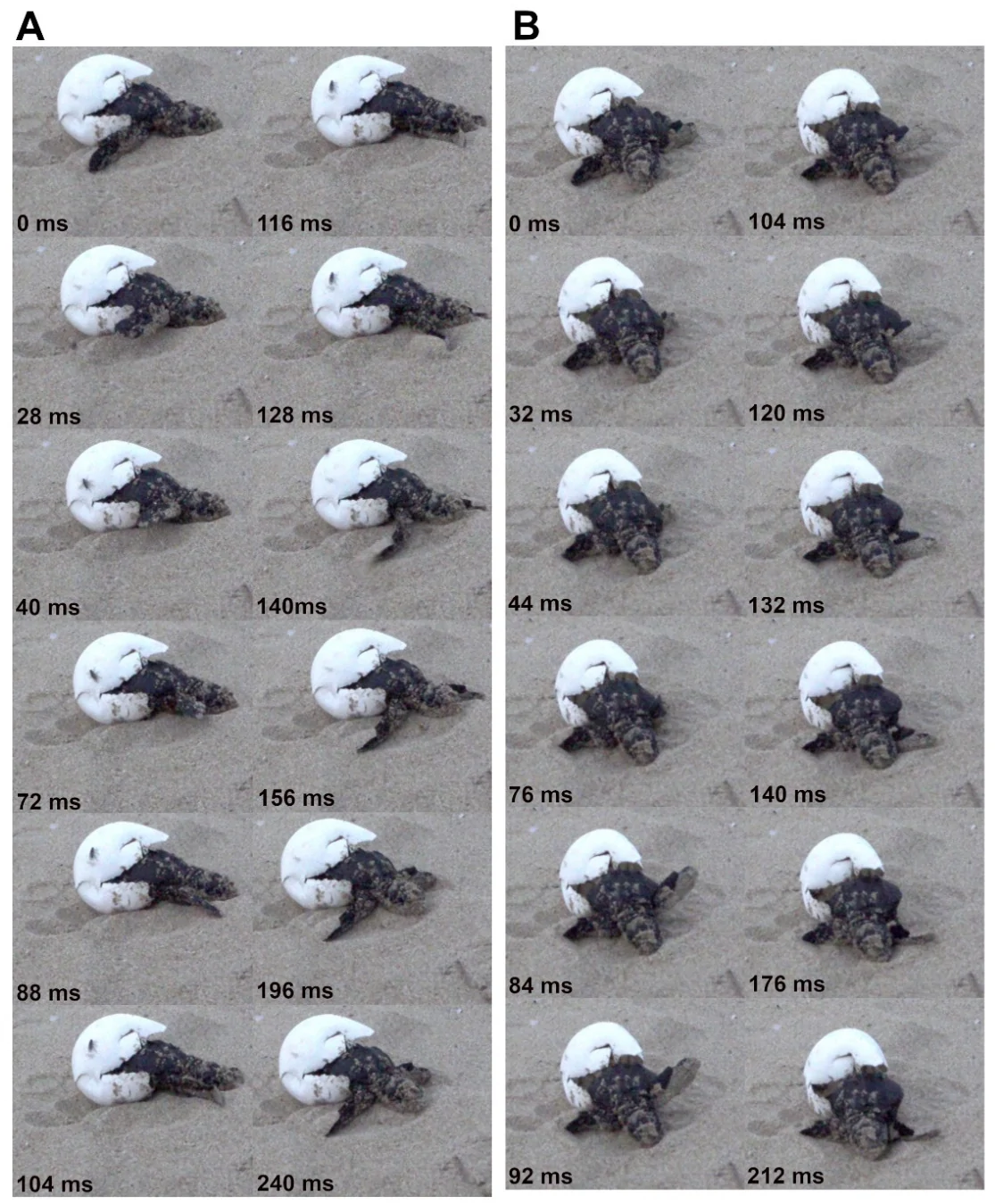
Representative terrestrial locomotor movements during emergence from the eggshell in hatchling *Caretta caretta*. (**A**) Sequence illustrating synchronous forelimb movements. One locomotor cycle comprises a stance phase (0–104 ms) followed by a swing phase (104–240 ms). (**B**) Sequence illustrating asynchronous forelimb movements, in which the left forelimb performs a swing phase while the right forelimb remains in contact with the substratum. The cycle comprises a first stance phase (0–76 ms), a swing phase (76–140 ms), and a second stance phase (140–212 ms). Photo credit: Florence Kirchhoff.

**Figure 4:**
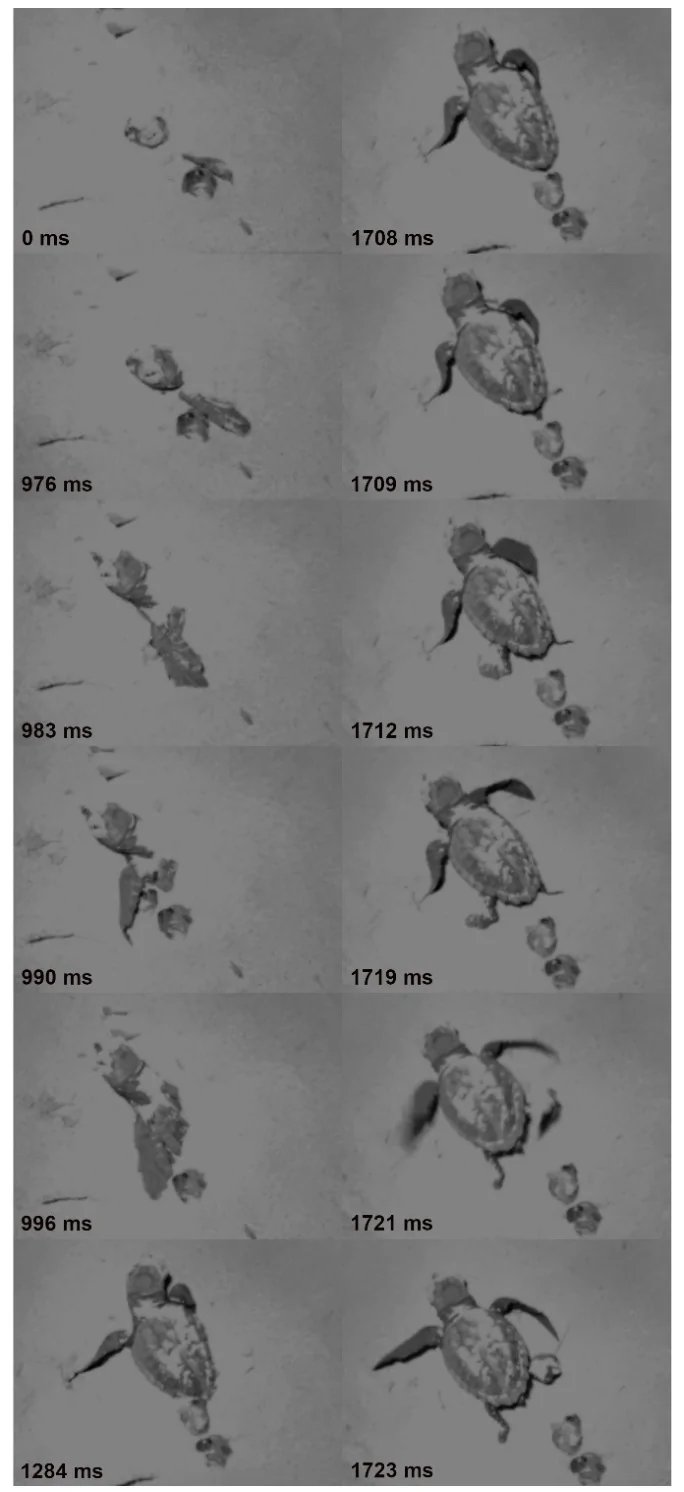
Representative fore- and hindlimb movements during nest emergence in *Caretta caretta*. A diagonal or diagonal-couplet pattern is used as soon as the hatchling emerges from the nest onto the sand surface. Photo credit: Florence Kirchhoff.

Hatchling sea turtles do not hatch synchronously, and early hatchlings can spend several days in the nest while sibling eggs continue to hatch before almost all hatchlings emerge as a single group, usually at night, within a vertical column (Supplementary Material File S2). This process is known as social facilitation [77] and has long been assumed to reduce predation. However, Rusli et al. [78] demonstrated that it also saves significant amounts of energy while reducing the time required to exit the nest. More recently, Ferrara et al. [79] showed that embryos within eggs and newly hatched turtles communicate acoustically, potentially contributing to the coordination of hatching and subsequent nest emergence. Our observations of *Caretta caretta* hatchlings revealed both coordinated synchronous and alternating forelimb movements during emergence from the eggshell (Figure 3). Coordination between the fore- and hind flippers was variable, with both synchronous and asynchronous movements being observed. During emergence from the nest, hatchlings predominantly used synchronous foreflipper movements once both forelimbs were released from the mechanical constraints imposed by the surrounding sand (Figure 4).

#### 3.1.2. Moving on Land

**Figure 5:**
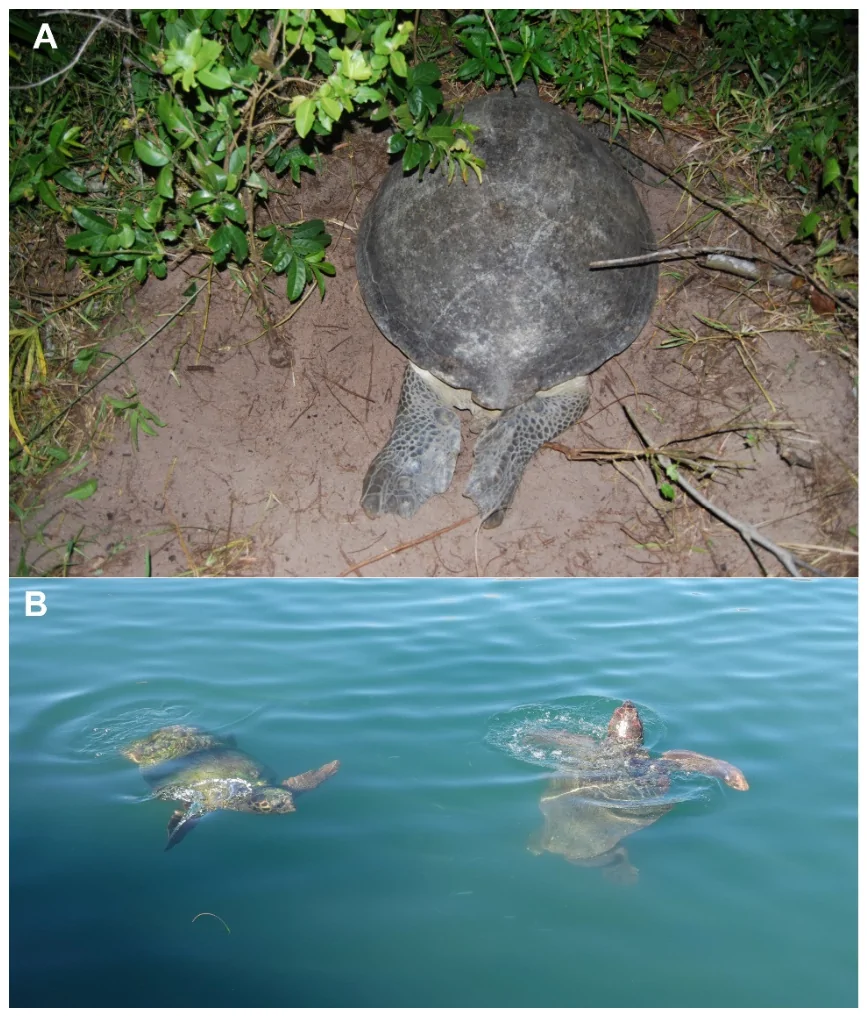
Representative locomotor activities in marine turtles showing the action of the hindlimbs. (**A**) Nesting female *Chelonia mydas* illustrating the use of the hindlimbs during nest excavation. (**B**) Two *Caretta caretta* swimming at Argostoli, Kefalonia, Greece, illustrating characteristic manoeuvring postures. Photo credit: Florence Kirchhoff.

All marine turtles use the terrestrial environment with highly variable properties for reproduction. Regardless of their phylogeny and their morphological and ecological traits, adult females must swim, leave the sea, move on to the beach, excavate a nest, deposit their eggs, and return to the sea (Figure 5). Hatchlings first emerge from the egg and then from the nest before moving towards the sea. The influence of substratum properties on locomotor performance differs between hatchlings and adult females because of their contrasting body size and mass. When moving on beaches, adults and hatchlings are in contact with sand, a granular solid material with various textures, porosities, and permeabilities that can also behave in a liquid-like manner [80,81].

During terrestrial locomotion, turtles encounter marked spatial variation in substratum properties, ranging from dry sand above the high-tide level through damp sand to water-saturated sand. The mechanical interactions between the limbs and the substratum continuously change according to substratum compaction, fluidity, and surface morphology. Experimental studies have shown that hatchlings moving over loose sand generate propulsion primarily through the forelimbs. During stance, the foreflippers penetrate the substratum, rotate to become nearly perpendicular to the direction of motion, and exploit local sand solidification to produce thrust while minimising slip. Therefore, propulsion results primarily from forelimb traction on the substratum, the hindlimbs mainly contributing to lifting and stabilising the body, with only a limited role in propulsion [82]. However, changing substratum conditions can strongly influence terrestrial locomotor performance and must therefore be considered when interpreting locomotor patterns.

#### 3.1.3. Repertoire of Terrestrial Locomotion

Table 1 summarises the terrestrial locomotor repertoire of extant sea turtles, distinguishing hatchlings from nesting adult females because these life stages experience markedly different biomechanical and ecological constraints. Unless otherwise specified, subsequent comparative analyses are performed at the species level, considering the terrestrial locomotor repertoire expressed across the life cycle (Supplementary Material File S1A). In all species, the limbs alone do not fully support the body during terrestrial locomotion by adult females or during nest emergence by hatchlings. Instead, the main support is provided by the ventral surface of the body (plastron). In all species, the limb cycle is divided into a stance (propulsion) and a swing (repositioning) phase. During strides, hindlimb movements during the stance phase ensure postural adjustments by partially elevating the turtle’s plastron from the sand to reduce friction and promote forward progression (Supplementary Material File S3).

**Figure 6:**
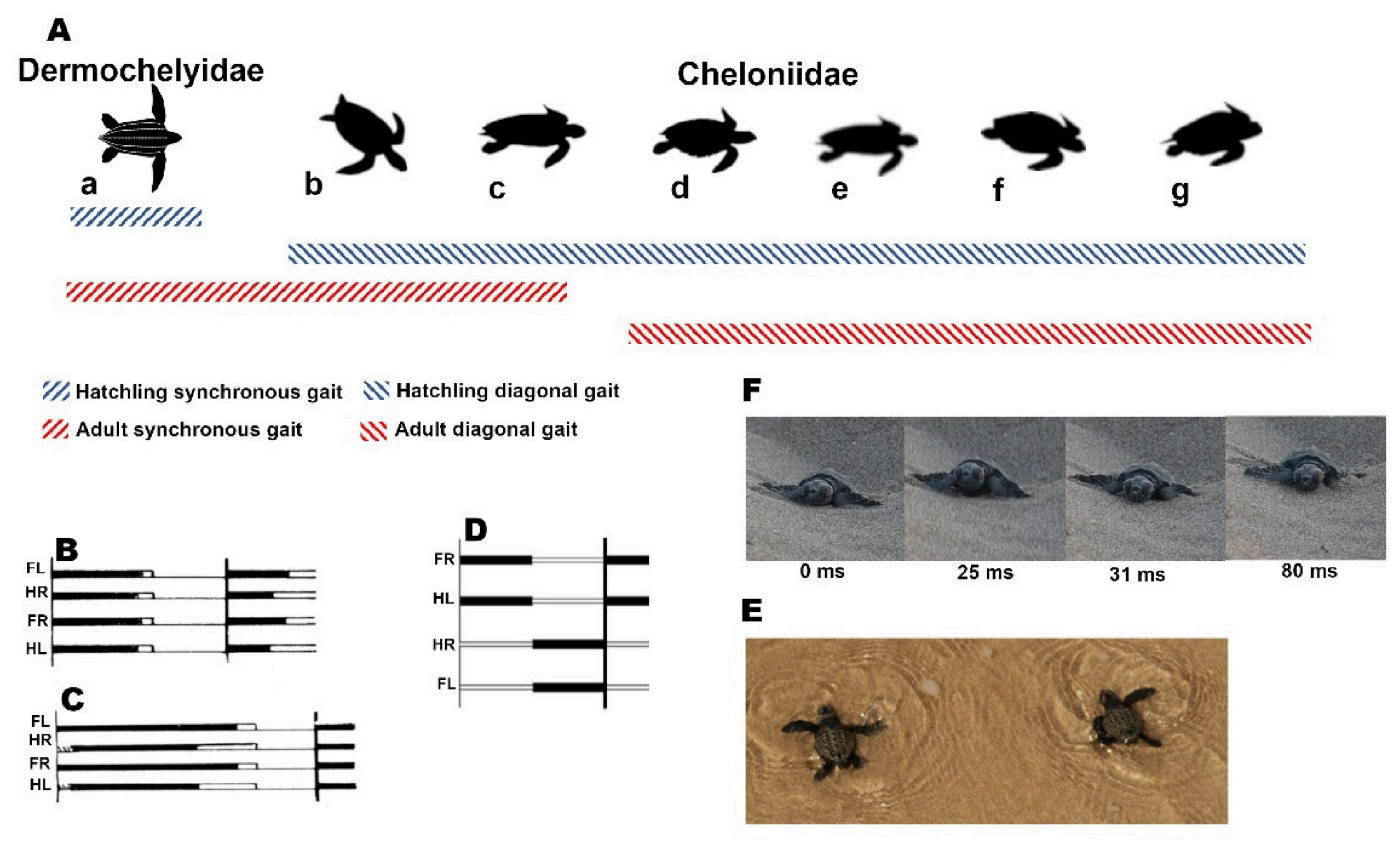
Comparison of terrestrial locomotor patterns in extant marine turtles. (**A**) Distribution of synchronous and diagonal gaits in hatchlings and adults of Dermochelyidae and Cheloniidae. (**B**) Representative gait diagram of a hatchling *Dermochelys coriacea*. (**C**) Representative gait diagram of an adult *D. coriacea*. (**D**) Representative diagonal gait with asynchronous limb cycles in a hatchling *Caretta caretta*. (**E**) Representative limb positions during diagonal gait as a hatchling *C. caretta* enters the sea. Photo credit: Julien Pfyffer. (**F**) Successive frames illustrating postural adjustments during synchronous foreflipper movements in a hatchling *C. caretta*. Photo credit: Florence Kirchhoff. Abbreviations: FL, left forelimb; FR, right forelimb; HL, left hindlimb; HR, right hindlimb. (**a**) *Dermochelys coriacea*; (**b**), *Chelonia mydas*; (**c**), *Natator depressus*; (**d**), *Eretmochelys imbricata*; (**e**), *Lepidochelys olivacea*; (**f**), *Lepidochelys kempii*; (**g**), *Caretta caretta*. Silhouettes of all species are from PhyloPic (https://www.phylopic.org/), and are available under the Public Domain Dedication (CC0).

During the stance phase, corresponding to the retraction phase that produces body elevation and forward thrust [83,84], each limb modifies and penetrates the substratum. Thus, when moving towards the water, two main types of limb motor coordination have been identified in hatchlings (Figure 6): (i) asymmetric locomotion with diagonal limb cycles (walking *sensu* Hildebrand-style gait diagram [85]), and (ii) synchronous limb cycles for postural adjustments.

In these turtles, synchronous forelimb cycles are used under various environmental constraints (e.g., dry sand) that limit the efficiency of diagonal limb cycles. In this case, both forelimbs are placed in similar anteriormost positions, and their retractions during the stance phase occur simultaneously to pull the turtle forward. This mode of locomotion is also used by *C. caretta* hatchlings to initiate a subsequent series of diagonal gait movements by elevating the anterior part of the body from the substratum when moving on damp sand immediately before reaching the water’s edge.

In diagonally coordinated locomotion, the body of a *C. caretta* hatchling follows a cyclic movement pattern involving elevation above the sand and forward displacement produced by the combined thrust of the hindlimbs and traction of the forelimbs on opposite sides of the body.

The body is elevated above the substratum to a variable extent and returns into contact with it at the end of the cycle [82]. During synchronous forelimb pulling in *D. coriacea*, elevation of the body occurs during the stance phase, corresponding to forelimb retraction relative to the body. In this case, the simultaneous movements of the forelimbs produce a similar effect combining the lifting and the forward movement of the body in a “crutching” manoeuvre as soon as the shoulder touches the sand [83,84].

**Figure 7:**
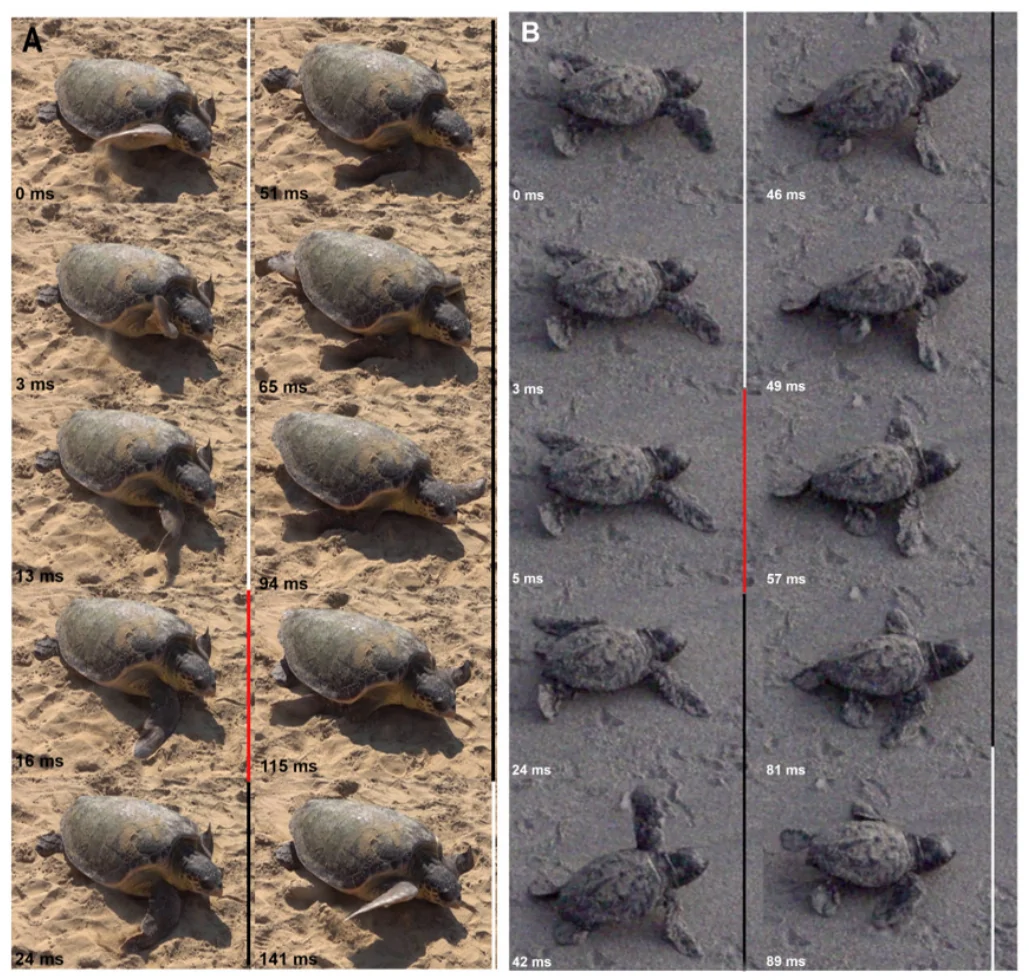
Representative limb cycles of *Caretta caretta* moving on sand. (**A**) Adult female moving on dry sand. (**B**) Hatchling moving on slightly damp sand. 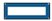 Swing phase. 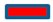 Frames of transition from swing to stance phase (first contact of the forelimbs on the sand). 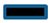 Stance phase. Photo credit: Florence Kirchhoff.

Regardless of species and life-history stage (adult female or hatchling), the forelimbs follow similar cyclic swing and stance phases. First, the limb is placed on the sand at the end of the swing phase and assumes an almost perpendicular position. Depending on sand compaction, the edge of the flipper penetrates the substratum to a variable depth, while the humeral surface makes contact with the sand (Figure 7A,B). As soon as the forelimbs are in this position, pushing associated with limb retraction begins. During pushing, the cutting radial edge of the forelimb exerts pressure on the sand to lift and move the turtle forwards. As shown in hatchling *C. caretta*, the net thrust forces generated remain below the yield force of the substratum, which responds differently according to its texture [81,82]. For example, damp sand becomes locally compacted behind the medial surface of the limbs, forming a ridge of variable length and height against which the turtle generates thrust during each stroke. This solidification does not occur when turtles move on compact sand because the limbs do not penetrate it significantly. During the swing phase associated with their protraction, the foreflippers are lifted and moved forward. When it reaches the maximal forward position, each humeral head rotates axially and sets its radial edge in contact with the sand.

**Figure 8:**
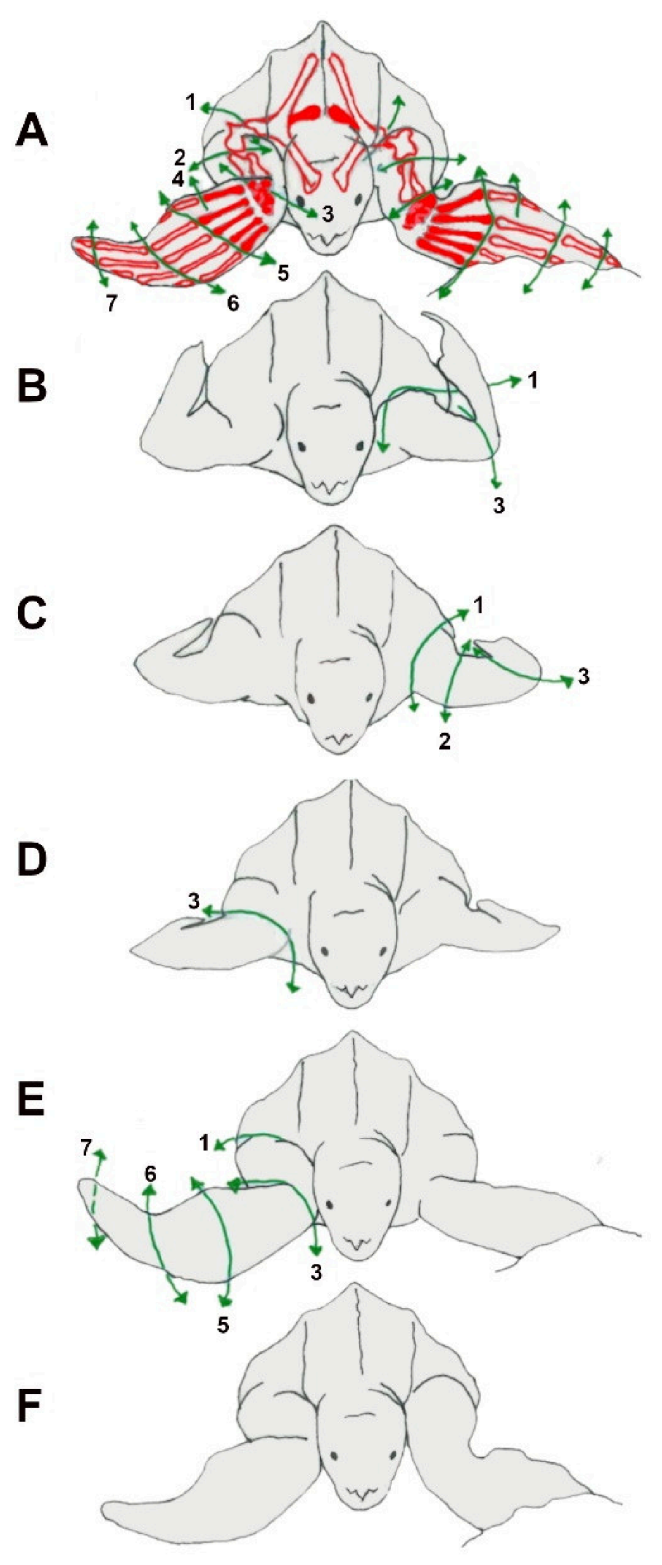
Representative front-view sequence of terrestrial locomotion in an adult female *Dermochelys coriacea*, showing the successive movements of the skeletal elements (red) during a complete foreflipper cycle. (**A**,**B**) Two frames showing hindlimb retraction during the stance phase, during which the turtle moves forwards. (**C**–**F**) Four frames showing the swing phase. Movements of the principal skeletal landmarks were reconstructed by skeletal manipulation and are indicated by green arrows. 1, Scapulocoracoid articulation; 2, glenohumeral joint; 3, humerus; 4, metacarpus; 5, joint between the metacarpus and proximal phalanges; 6, joint between the proximal and distal phalanges; 7, distal phalanges. Photo credit: Sabine Renous (used with permission).

In *D. coriacea*, the vertical rising of the body occurs during the thrust produced by the forelimbs, associated with simultaneous retraction of the limbs (Figure 8). In adult turtles using this synchronous pattern, the coordinated actions of the fore- and hindlimbs probably combine body elevation with forward progression during the stance phase. Forward acceleration coincides with contact between the humeral region and the sand. The turtle then progresses at a relatively constant speed before the body is lowered at the end of the stance phase.

#### 3.1.4. Locomotor Evolution

**Figure 9:**
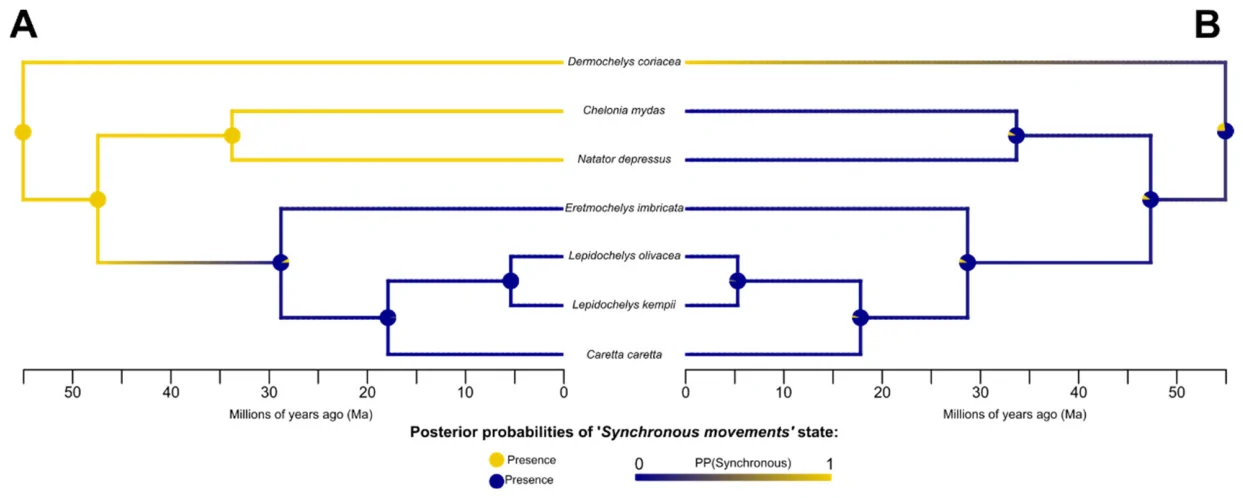
Phylogenetic comparative analysis of turtle locomotor behaviours. Ancestral-state reconstruction of synchronous movements (present/absent) in adults (**A**), based on the constrained “Synchronous → Asynchronous” model. Reconstruction for hatchlings (**B**), based on the Equal-Rates model. Pie charts at each node represent posterior probabilities from stochastic character mapping (*n* = 100), and the branch colour gradient indicates the posterior density of the occurrence of synchronous movements.

Because locomotor behaviour differs between hatchlings and nesting females, ancestral-state reconstructions were performed separately for each life stage before being interpreted within the species-level comparative framework adopted in this study. In adult turtles (Figure 9A), ancestral-state reconstruction was based on a comparison of the four evolutionary models described in the Section 2. The unidirectional *Synchronous → Asynchronous* model received the best support (AICc = 10.18; BIC = 9.33), closely followed by the Equal-Rates (ER) model (AICc = 10.27; BIC = 9.42). The *Asynchronous → Synchronous* model (AICc = 10.85; BIC = 9.99) and the All-Rates-Different (ARD) model (AICc = 14.42; BIC = 11.31) performed more poorly. Although differences in the AICc and BIC between the two best models were minimal, the *Synchronous* → *Asynchronous* model was selected for illustration as a directional and parsimonious scenario, while recognising that its support over the ER model was negligible.

Under this model, stochastic character mapping revealed an average of 1.09 transitions across the phylogeny (*n* = 100 maps). Lineages spent approximately 63% of their evolutionary history in the synchronous state and 36% in the asynchronous state. In hatchling turtles (Figure 9B), model comparison favoured the Equal-Rates (ER) model, which showed the lowest AICc (8.72) and BIC (7.87). The unidirectional models *Synchronous → Asynchronous* (AICc = 8.87; BIC = 8.02) and *Asynchronous → Synchronous* (AICc = 8.90; BIC = 8.05) received slightly higher scores, but the differences remained minimal, indicating that the data did not clearly discriminate among these models.

**Figure 10:**
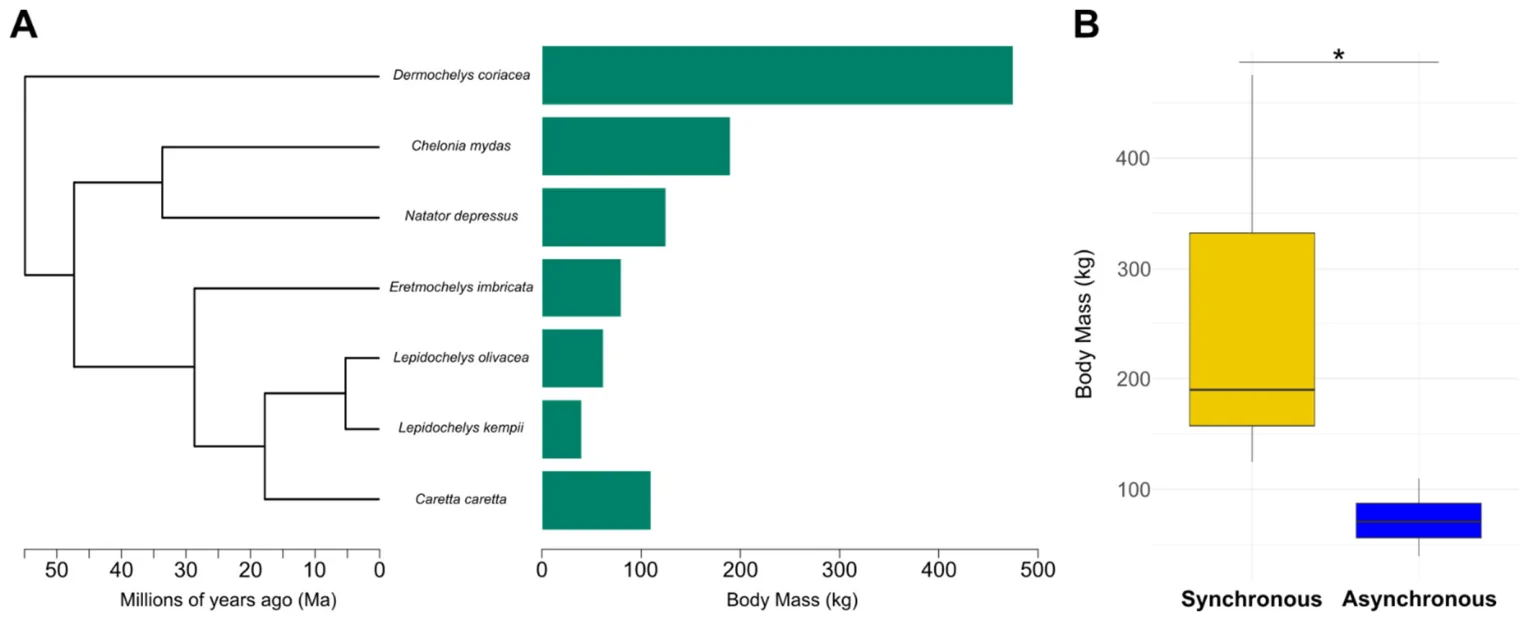
Comparative analysis of locomotor synchrony and body mass in adult marine turtles. (**A**) Phylogenetic signal in body mass assessed using Blomberg’s *K*. (**B**) Comparison of body mass between adult marine turtles exhibiting synchronous and asynchronous locomotion using a Mann–Whitney *U* test (*p* < 0.05).

Given its simplicity and balanced fit, the ER model was selected for illustration (Figure 9B). Under this model, stochastic character mapping inferred an average of 4.69 state transitions across the phylogeny, including approximately 2.23 from synchronous to asynchronous and 2.45 from asynchronous to synchronous. The reconstructed character evolution indicated that lineages spent approximately 18% of their evolutionary history in the synchronous state and 81% in the asynchronous state, indicating a predominance of the asynchronous condition in hatchlings. The analysis of phylogenetic signals for adult turtle mass revealed a significant signal (K = 1.87, *p* = 0.02; Figure 10A), suggesting that closely related species tend to resemble each other in body mass more than expected under a Brownian motion model of evolution. This supports the idea that mass is phylogenetically conserved in this group. Additionally, a Mann–Whitney U test revealed a significant difference in body mass between species with different locomotor states: asynchronous species were significantly lighter than synchronous ones (U = 12, *p* < 0.05, Figure 10B).

### 3.2. Penguins

Birds are flying theropod dinosaurs. Typically, birds exhibit bipedal terrestrial locomotion with alternating movements of the hindlimbs, expressed as walking or running, combined with synchronous flapping of the wings during take-off and flight [86,87]. Variations in hindlimb coordination occur, notably during hopping in many passerines [87]. No aquatic bird lives their whole life in water; they all must return to land to breed. Most types of water bird show relatively little modification for the aquatic environment beyond webbed feet, large uropygial (preen oil) glands, and adapted beak structures. However, some species are adapted for swimming and diving below the water surface to capture prey. Plunge-divers (e.g., gannets, pelicans, terns, kingfishers) dive into the water from height to capture fish that have been spotted from the air. Typically, they have relatively long wings and modified necks, skulls, and eyes [88,89], but their terrestrial locomotion is relatively uncompromised, except in the case of kingfishers (which have unwebbed feet modified for burrow digging).

Most pursuit-diving birds, including cormorants, auks, diving petrels, loons, grebes, and penguins, have fusiform bodies with relatively high fineness ratios, which reduce drag during submerged swimming [90,91]. This streamlined body form occurs whether submerged propulsion is generated primarily by the feet, as in grebes, loons, and cormorants, or by the wings, as in penguins, auks, and diving petrels. Their legs, often short, are mounted posterior to the centre of mass when submerged. On land, their relatively short hindlimbs limit stride length, and the posterior position of the feet requires an upright posture to maintain the centre of mass above the supporting base. Such walking is relatively unimportant in flying birds as they can fly close to nests or burrows, so do not have to walk long distances.

**Figure 11:**
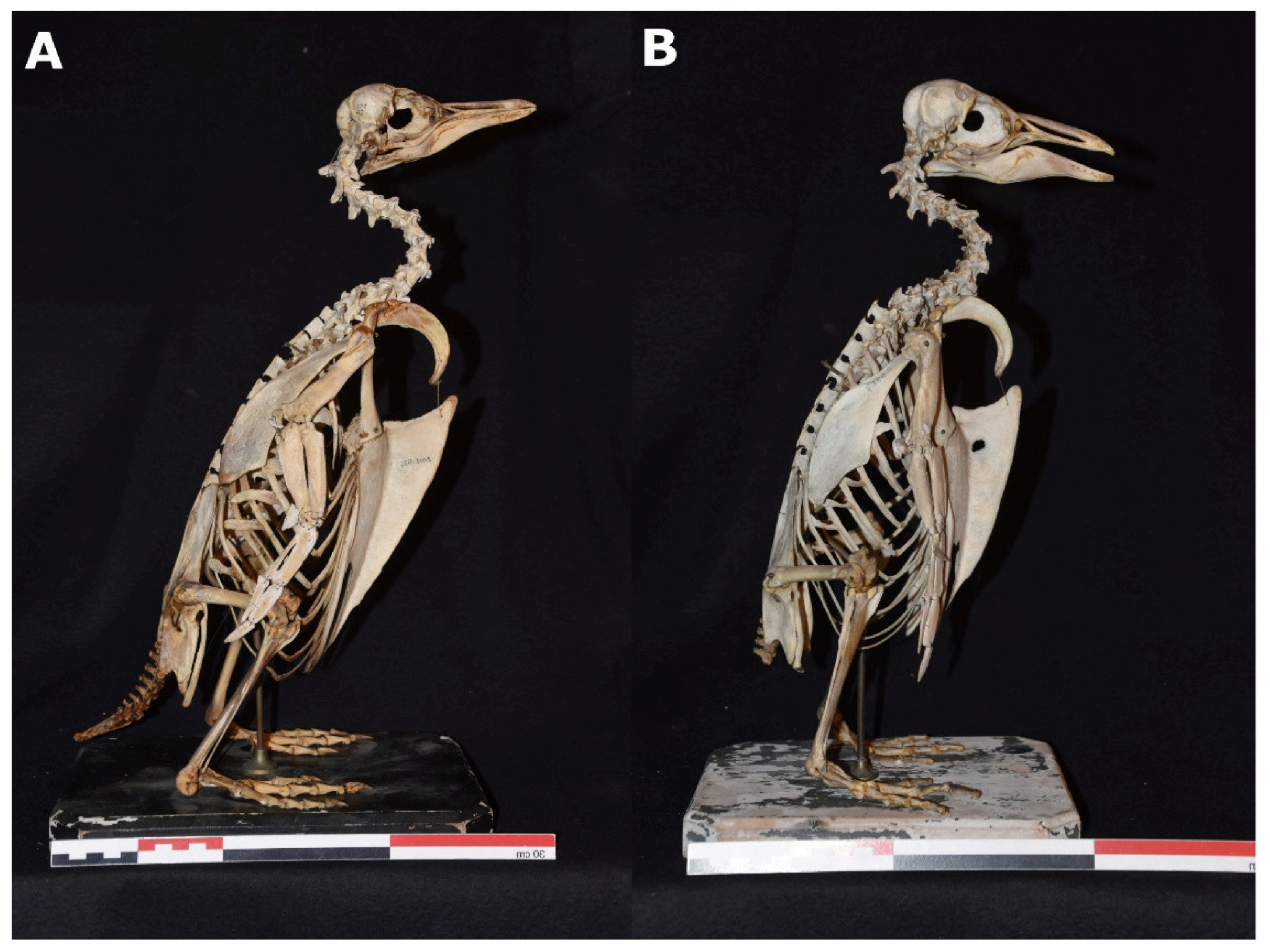
Comparative skeletal morphology of two penguin species with contrasting terrestrial locomotor behaviours (Muséum national d’Histoire naturelle, Paris, France). Both species differ in their usual mode of terrestrial progression. (**A**) The king penguin, *Aptenodytes patagonicus*, progresses on land primarily by bipedal walking and, when necessary, jumping; routine hopping has not been reported. (**B**) The macaroni penguin, *Eudyptes chrysolophus*, commonly uses hopping as a routine mode of terrestrial locomotion. Thus, the contrast in locomotor behaviour is not clearly associated with an obvious difference in gross skeletal organization visible at this scale Scale bar (red bar) = 30 cm. Photo credit: Eric Pellé.

Penguins (Spheniscidae) are diving, flightless birds whose wings are modified to act as lift-generating flippers; they spend much of their lives at sea, swimming long distances in pursuit of prey. Their skeletal structures are dense and robust, helping them withstand the mechanical loads associated with aquatic locomotion [92] and reduce buoyancy. For example, the comparison between *Aptenodytes patagonicus* and *Eudyptes chrysolophus* illustrates that differences in terrestrial locomotor modes may occur despite a broadly similar general skeletal organization (Figure 11). The figure is illustrative and does not constitute a formal morphometric comparison. Penguin feathers are also unusual. First, feathers are present over the entire body surface rather than occurring in tracts separated by bare skin, as in most other bird species. Next, each feather has two parts: an anterior flattened element that provides the smooth, waterproof outer covering on the penguin’s body surface, and a posterior down-like after-feather that provides highly efficient insulation [93]. These relationships were investigated quantitatively and documented much later.

Available evidence concerning relationships amongst extant and extinct penguin species indicates that all extant penguin species are relatively young in terms of geological time [94,95]. The oldest recognisable penguin fossils date from approximately 61 Ma, as documented by the early diversification of these birds [96,97]. It is accepted that they evolved from flying ancestors and are monophyletic; their nearest extant relatives have long been believed (on morphological grounds) to be other marine birds (either Procellariiformes, Gaviiformes, or Pelecaniformes; see [97]) that fly. However, early molecular studies suggested that their nearest relatives might possibly be storks (Ciconiidae) [98]. More recent molecular studies have restored Procellariiformes (fulmars, albatrosses, petrels, storm petrels, and shearwaters) as the sister group of penguins [99].

#### 3.2.1. Moving on Land

Penguins originally evolved in temperate rather than cold waters [100], and the number of recognised extant species ranges from 16 to 19 depending on taxonomic interpretations, particularly those concerning rockhopper penguins [101]. Geographically, they range from the equatorial Galapágos Islands in the north to 77° S on the stable pack ice of Antarctica. Though homogenous in anatomy, they vary widely in size, from the 0.33 m tall Little penguin (*Eudyptula minor*) with a body mass of 1.5 kg, to the 1 m tall, 22–45 kg Emperor penguin (*Aptenodytes forsteri*). Most species are found in cold/warm temperate areas, and although they cover long distances (10 s to 100 s of km) by swimming to forage, they do not need to move more than a few hundred metres between the sea and their breeding sites on land. A few species found in the Subantarctic and Antarctic undertake much more substantial terrestrial migrations over snow or pack ice to reach their breeding areas. Adélie (*Pygoscelis adeliae*), Chinstrap (*Pygoscelis antarcticus*), and Gentoo (*Pygoscelis papua*) penguins all breed (in whole or part of their range) in ice-free areas that require crossing over several km of sea ice or snow-covered land to reach them. Adélie penguins make terrestrial treks of 100 km or more [102]. The Emperor penguin is the only penguin species that breeds on stable pack ice or ice shelves, and can trek <120 km across ice and snow to reach breeding areas; females return to the sea after laying eggs that are incubated by the males, so they accomplish a round trip of <240 km.

**Figure 12:**
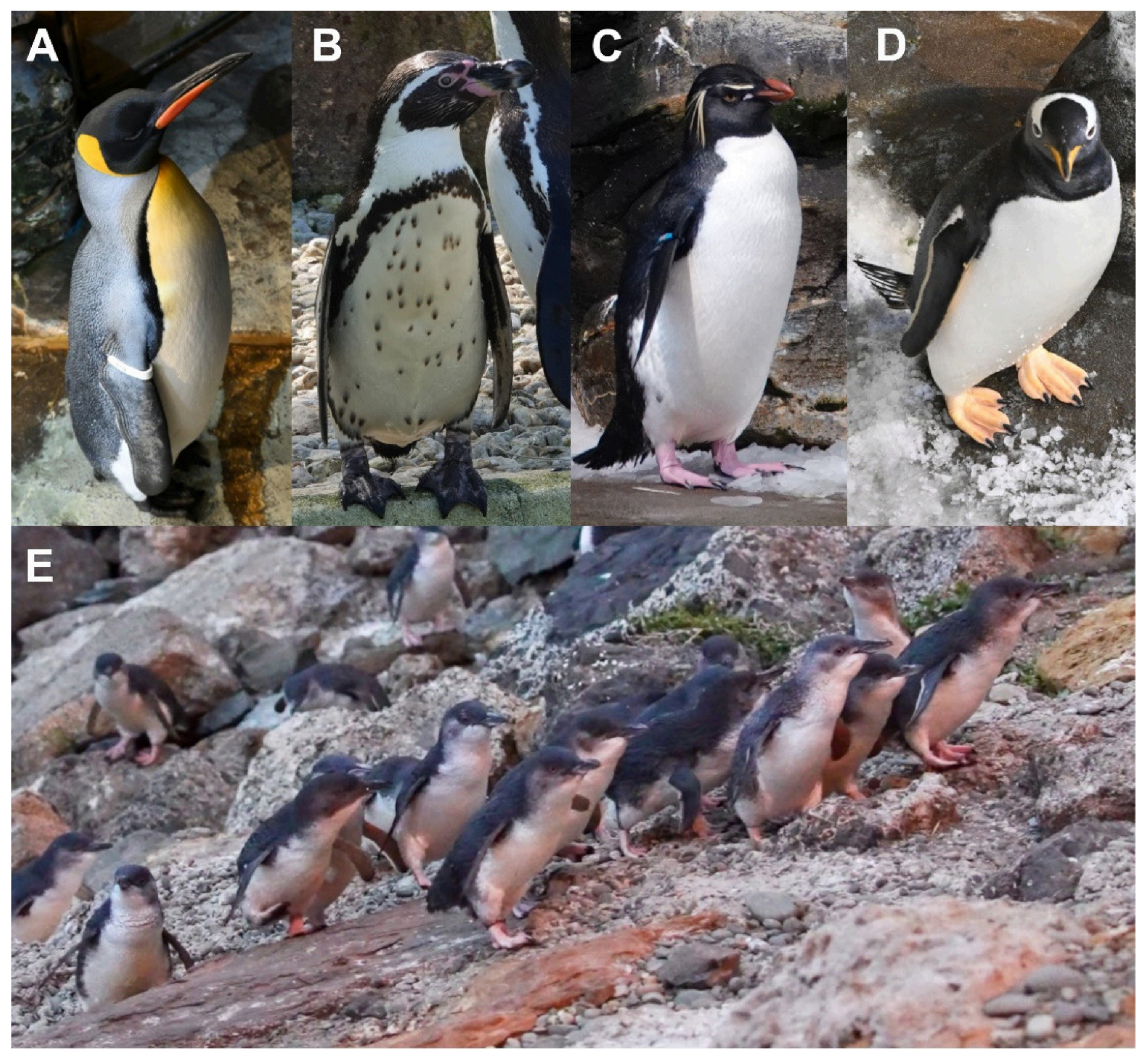
Representative locomotor postures and patterns. (**A**–**E**) Postures of five penguin species of different sizes: (**A**) King penguin, *Aptenodytes patagonicus*; (**B**) Humboldt penguin, *Spheniscus humboldti*; (**C**) Rockhopper penguin, *Eudyptes chrysocome*; (**D**) Gentoo penguin, *Pygoscelis papua*; and (**E**) Little penguin, *Eudyptula minor*. (**A**–**D**) Photo credit: Florence Kirchhoff. (**E**) Photo credit: Philippa Agnew.

Penguins use a variety of postures and modes of terrestrial locomotion (Figure 12; Supplementary Material File S1B) that include walking, jumping, hopping, and tobogganing [101,102,103,104]. The hindlimb skeletal and muscular morphology of penguins has attracted much study (e.g., [105,106]). It should first be recognised that the legs of penguins, though short by comparison with many other birds (especially in large King and Emperor penguins), are rather longer than they appear externally. The short femurs and kneecaps are covered by skin, whereas the belly and plumage obscure much of the longer tibiotarsi so that only the feet, ankles, and tarsometatarsi are visible in a standing penguin. Each padded foot has three large, webbed, and clawed toes, plus a small, rearward-directed clawed hallux. The limb bones are much larger and denser than those of flighted birds [106]. Femurs are notably thick-walled [91].

**Figure 13:**
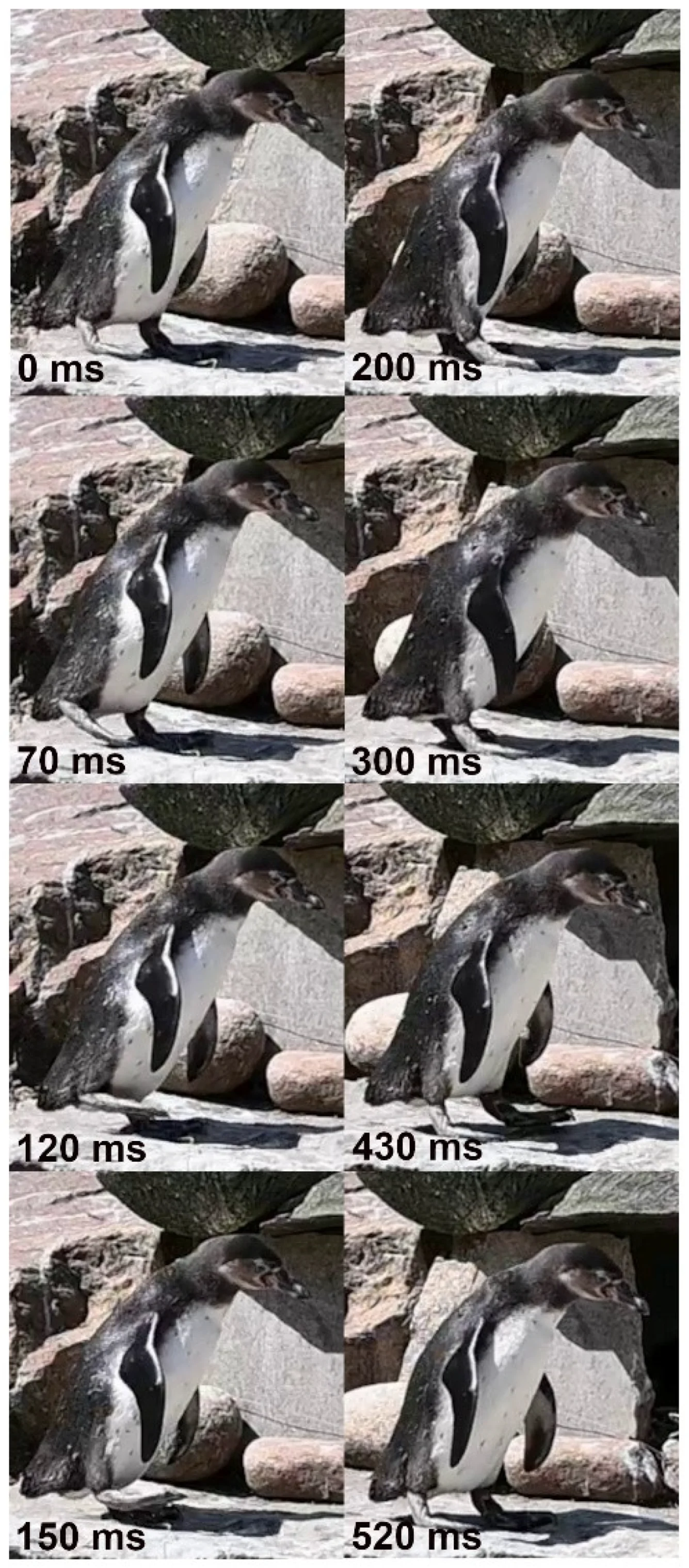
Representative walking sequence of *Spheniscus humboldti* on a solid substratum, illustrating the alternation of swing and stance phases during bipedal locomotion. The locomotor cycle of the left hindlimb comprises a swing phase (0–200 ms) followed by a stance phase (200–520 ms). Photo credit: Florence Kirchhoff.

The characteristic mechanism used during slow walking is known as ‘waddling’, and involves a near-upright posture, large lateral displacements of the body in the frontal plane during each stride and outwardly directed feet (Figure 13; [107]). Effectively, the penguin functions like an inverted pendulum, rocking from side to side in the frontal plane during each stride. As the bird rocks back towards its medial position, the right foot is placed on the substratum.

**Figure 14:**
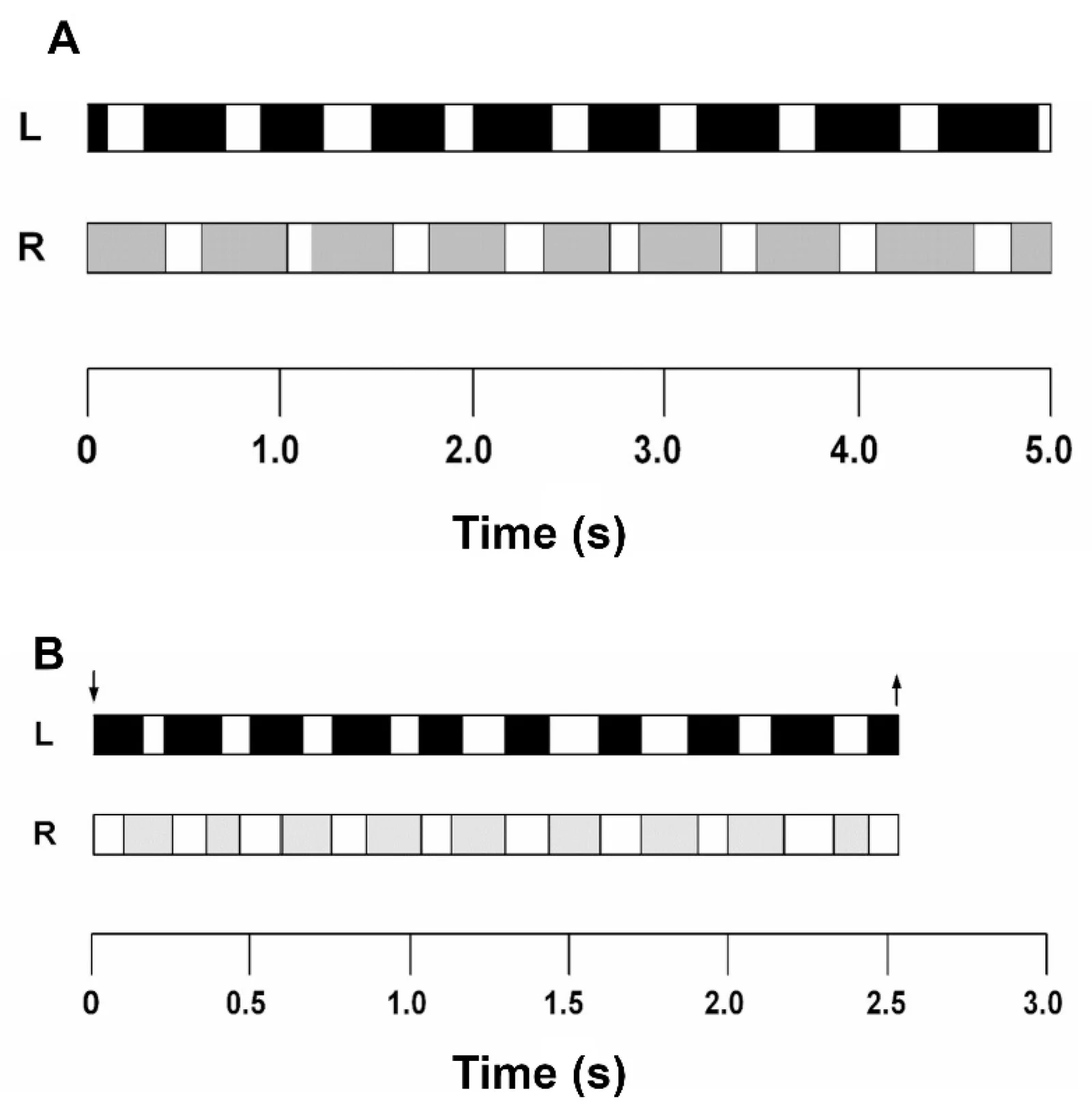
(**A**) Sequence (duration 5 s) of slow walking (waddling) in an Adélie penguin (*Pygoscelis adeliae*). L= left foot, R = right foot. White areas of the horizontal bars represent periods when the feet are elevated above the ground; black and grey areas represent periods when the left and right feet (respectively) are in contact with the substratum. Stride frequency = 1.8 strides s^−1^. Duty factor (mean value of the two feet) = 0.68. (**B**) Sequence of fast walking in an Adélie penguin (*Pygoscelis adeliae*). L, left foot, R, right foot. White areas of the horizontal bars represent periods when the feet are elevated above the ground; black and grey areas represent periods when the left and right feet (respectively) are in contact with the substratum. Down arrow indicates point at which the left foot was placed on the substratum (thin snow over sea ice); up arrow indicates point at which the left foot left the camera view. Stride frequency = 3.7 strides s^−1^. Duty factor (mean value of the two feet) = 0.57.

As the bird rocks back towards its medial position, the right foot is placed on the substratum. As rocking continues to the right, the left foot is raised and brought forward; as the body returns towards its medial position, the left foot is placed on the substratum, and the alternating sequence continues (Figure 14). A corresponding inverted pendulum operates (at lower amplitude) in the sagittal plane; penguins tend to lean slightly forwards and extend their foreflippers to maintain stability and potentially enhance the pendular exchange of mechanical energy. Waddling is not unique to penguins; it is also found in ducks, cormorants, petrels, and albatrosses.

Pinshow et al. [102] studied the energetics of waddling of Little, Adélie, and Emperor penguins. They found that the energetic costs of such walking were significantly higher in penguins than in other birds or quadruped mammals of similar body mass, largely because stride frequencies required to sustain a given walking speed were much higher in penguins because of their short legs/strides. The relative inefficiency of the side-to-side nature of waddling was implied. These authors suggested that high walking speeds were unnecessary in penguins because their island or high-latitude breeding areas featured no natural terrestrial predators. This is a dubious proposition, as penguins originated in temperate areas [100] where terrestrial predators are common. Extant penguins living on mainland South America and South Africa are preyed upon by foxes, mongooses, and pumas, whereas Adélie and King penguins are preyed upon by skuas and giant petrels that often attack whilst walking/jumping, behaving as surrogate terrestrial predators. Griffin and Kram [108] demonstrated (in Emperor penguins) that side-to-side motion during waddling was highly efficient during slow walking (at about 0.5 m s^−1^, a common natural velocity). Lateral movements increased the kinetic energy available to convert into gravitational potential energy. Overall, waddling penguins slowly recovered about 80% of the mechanical energy of each stride. These authors concluded that the energetic cost of waddling results from the high stride frequency, which requires the use of faster muscles that are less efficient than slow-twitch muscles, rather than from excessive movements in the frontal plane. Kurz, Fisher, and Gates [109] later demonstrated that side-to-side movement in waddling penguins also increased stability and resistance to falling, contrary to expectations derived from studies of humans.

**Figure 15:**
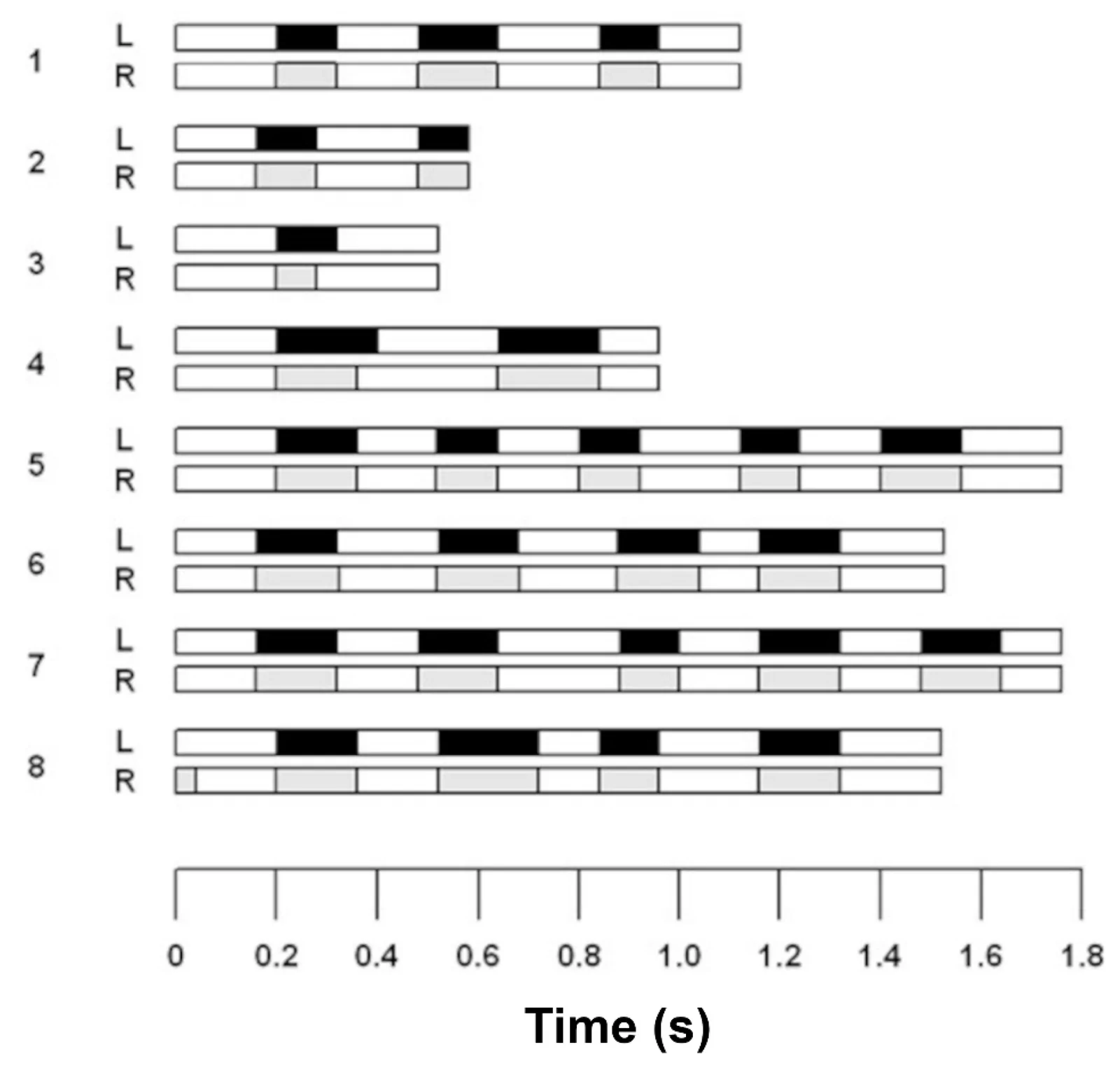
Eight sequences (order 1–8) derived from a single hopping episode by a northern Rockhopper penguin (*Eudyptes moseleyi*) held at Edinburgh Zoo in 2018 (see https://www.youtube.com/watch?v=z0rJNDmtGGw URL last accessed on 6 October 2026). L = left foot, R = right foot. White areas of the horizontal bars represent periods when the penguin was totally elevated above the ground; black and grey areas represent periods when the left and right feet (respectively) were in touch with the substratum. Between the sequences, the penguin was either out of camera view or the feet were obscured. In most sequences, take-off and landing of the feet were closely synchronised (within 0.04 s) throughout. In sequence 3, the right foot landed slightly before the left foot; in sequence 8, the first take-off was slightly unsynchronised, and thereafter the hops were in synchrony. Descriptive analysis (*n* = 28 hops) shows that the mean time in air (left foot) was 0.178 s vs. 0.176 s for the right foot; the mean time on the ground (left foot) was 0.145 s vs. 0.144 s for the right foot. Hopping frequency (based on elapsed time between take-offs) was therefore 3.1 hops s^−1^. Mean duty factors were calculated for sequences 1–4 (0.425) and 5–8 (0.466), showing a slight increase in duty factor during the second half of the hopping episode.

Several penguin species (including Little, Chinstrap, Gentoo, and Adélie) are capable of briefly walking much faster with little or no waddling. Such walking has attracted little study. We conducted a preliminary investigation of fast walking in an Adélie penguin (see https://www.youtube.com/watch?v=y18PU3uKRAs URL last accessed on 6 October 2026). It was only possible to analyse gait by identifying the timing of the raising and lowering of the feet, though qualitative information about posture is shown in Figure 12. The Adélie moved at about 3.7 strides s^−1^; such a high stride rate is to be expected as characteristic of large, heavy birds [110]. The observed gait pattern suggests that the penguin was walking close to the transition to running, because there was often minimal overlap between the periods of contact of the two feet. However, its duty factor was 0.57, indicating walking rather than running [111]. There is little or no evidence that any penguin species can run on level ground, although Rockhopper penguins sometimes run briefly downhill. It is also evident that fast-walking Adélie penguins show limited movement in the frontal plane as the sagittal stride length increases. The foreflippers are also not extended laterally but held behind the body (Figure 12 and Figure 15).

The phylogenetic distribution of hopping in penguins has not been previously investigated. The ability to hop is based on the ability to jump—effectively, hops are repeated jumps. All penguins can jump, although King and Emperor penguins can only manage a height of a few cm (except when jumping out of water, propelled by fast swimming). We inspected publicly available videos to identify those species that were able to hop (Table 2); they all belonged to two genera—*Pygoscelis* and *Eudyptes.* Hopping is better developed in *Eudyptes*, which is a very recent genus [94]. Rockhopper and Macaroni penguins can travel for tens/hundreds of metres by hopping, whereas Gentoo, Adélie, and Chinstrap penguins use hopping over much shorter distances, especially the large Gentoo. We evaluated the footfall of hopping in a northern Rockhopper *Eudyptes moseleyi* and found that it involved in-phase use of the hind legs and a duty factor <0.5.

**Figure 16:**
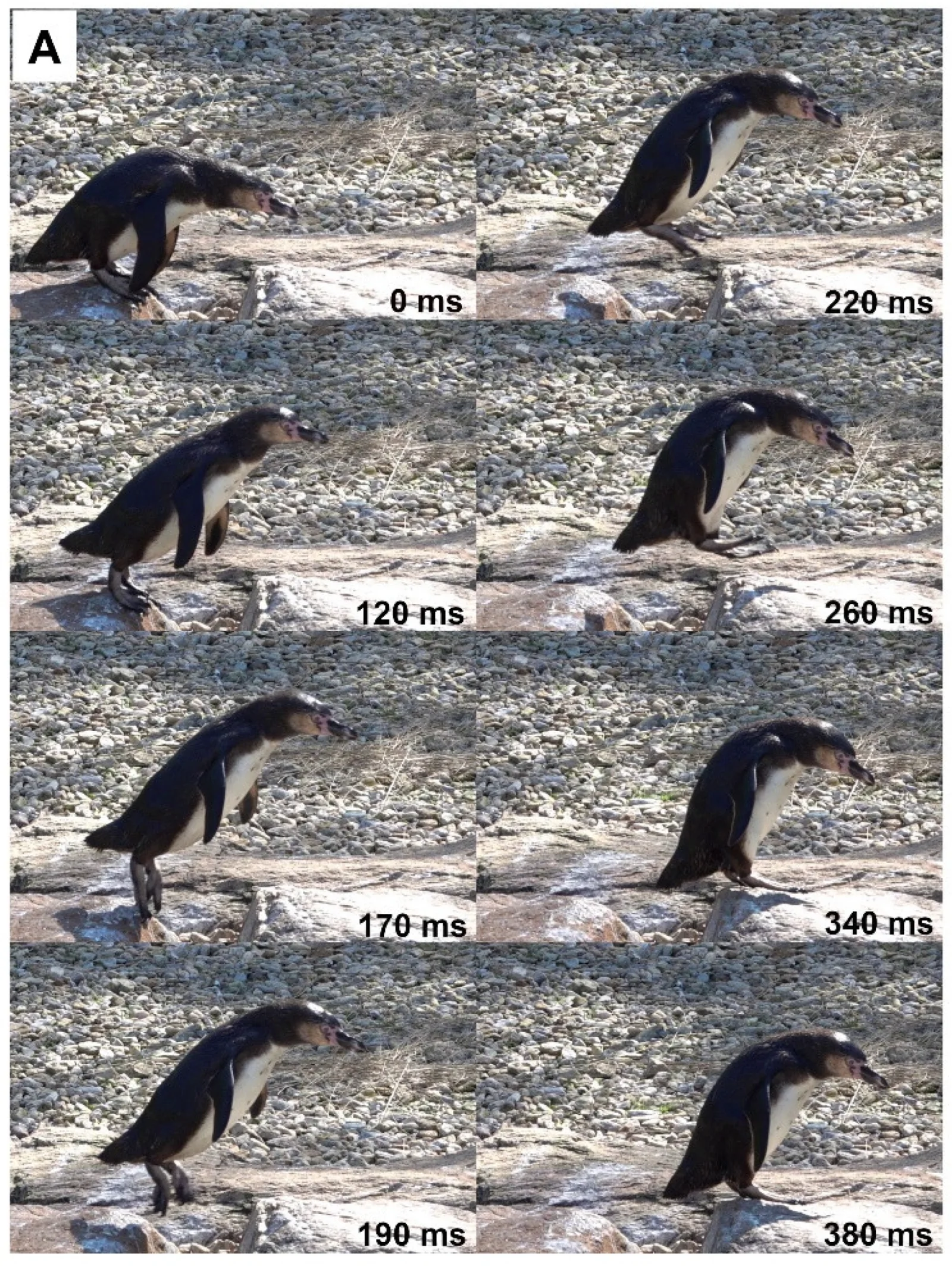

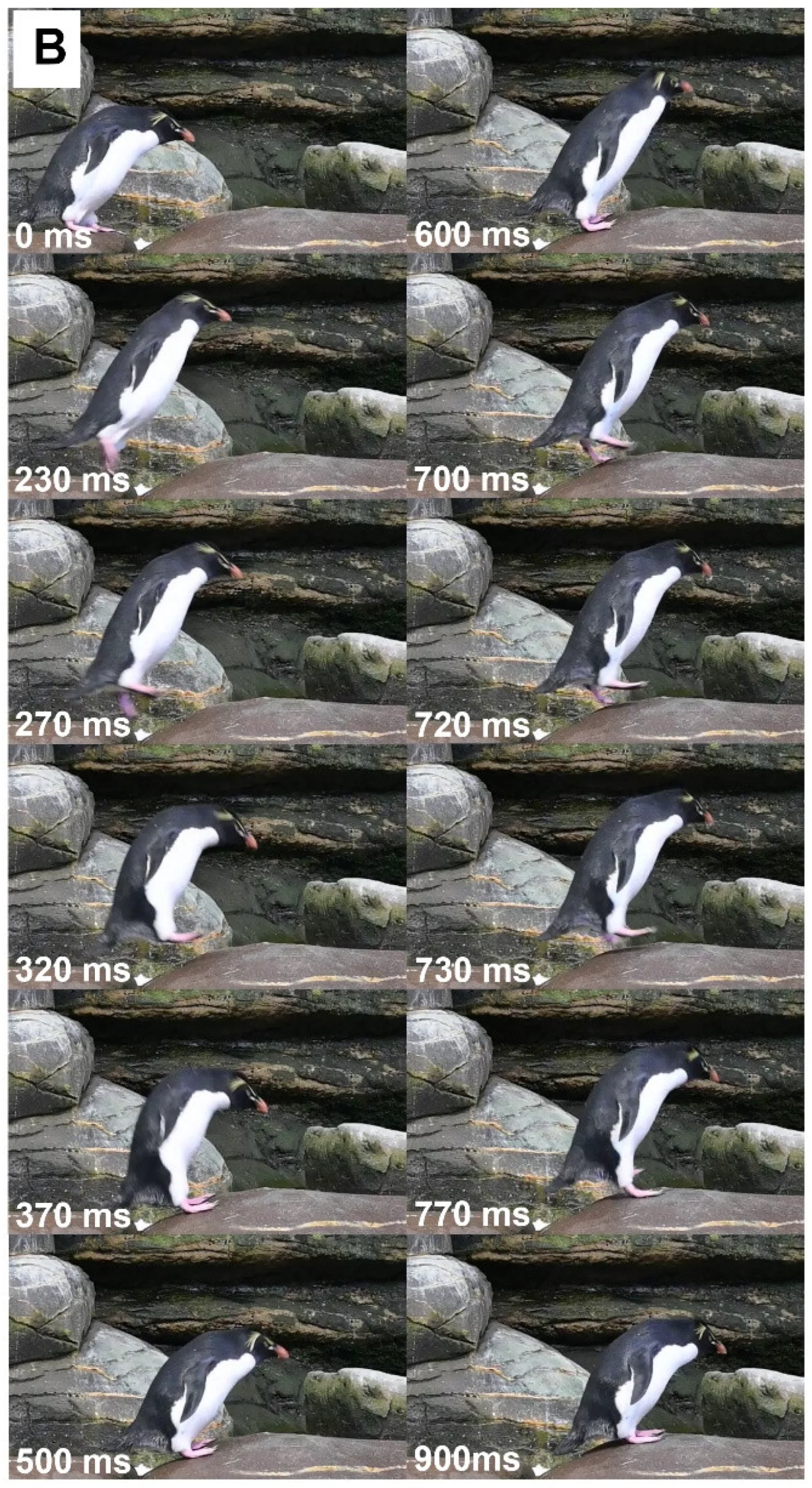
(**A**) Typical jumping sequence in *Spheniscus humboldti*. (**B**) Typical hopping sequence in *Eudyptes chrysocome*. The hopping sequence comprises two successive jumps, with the second take-off beginning immediately after completion of the first landing. Photo credit: Florence Kirchhoff.

Hall, Codd, & Nudds [112] have recently investigated the wing and leg musculature of Macaroni penguins (*E. chrysolophus*). They describe a pair of unique muscles (each named ‘adductor tibialis’) that connect contralateral tibiotarsii (via slight connections to the sternum). They effectively tie the legs together when they contract, helping jumping and hopping, but restricting walking. It is presently not known whether other hopping species have such muscles, though Macaroni and Rockhopper penguins are congeneric (see above). More anatomical studies are desirable because jumping is one of the locomotor modes used by penguin species of various sizes (*Pygoscelis* sp., *Eudyptes* sp., *Spheniscus* sp., and *Eudyptula* sp.). Jumping is divided into three phases: take-off with synchronous feet impulsion, flight through the air, and landing, as shown in a typical sequence of a single jump by *S. humboldti* (Figure 16A) and in a series of jumps (i.e., hopping) by *E. chrysocome* (Figure 16B). In penguins, no wing movements accompany preparation or jumping for a height between the take-off and landing platforms less than 30% of the body length of the animal.

It has been known since the 19th century that Adélie, Chinstrap, Gentoo, and Emperor penguins use an alternative and novel mechanism of locomotion on snow and ice—‘tobogganing’—in which the birds lie on their ventral surfaces and propel themselves quickly by alternate action of their feet. The term ‘toboggan’ is derived from ‘topaĝan’, a Mi’kmaq First Nations word that describes a broad piece of wood, curved upwards at the front, used extensively for centuries in Canada to carry goods or people when towed over snow. Toboggans were coated with waterproof materials (e.g., pine pitch and animal fat) that also created a hydrophobic surface to minimise friction between the snow and the toboggan. Penguin plumages in cold-water species have hydrophobic outer surfaces coated with preen oil that also inhibits ice-crystal formation [113], so the bellies of these penguins have low friction levels when the birds are tobogganing.

The definitive study of penguin tobogganing is that conducted by Wilson et al. [103]. They studied Adélie penguins on the Antarctic Peninsula and showed that tobogganing was always quicker than walking, especially downhill. Tobogganing was particularly efficient in soft snow; walking penguins sank and had to pull their feet out of the snow at each step. In contrast, tobogganing penguins rode over the snow surface and did not sink. They observed a high incidence of walking in penguins moving over hard snow, but all Adélie penguins used tobogganing over patches of soft snow. A recent (2019) film of Emperor penguins moving across flat, snowless sea ice against a strong wind shows 32 (84%) of 38 penguins tobogganing (see https://www.youtube.com/watch?v=9dvipJ1vEyk URL last accessed on 6 October 2026), indicating that a greatly reduced frontal area when moving by tobogganing may also be important in exposed areas.

Until recently, it was assumed that tobogganing was a specialised form of bipedal locomotion. However, several species of penguin are capable of true quadrupedal locomotion, in which all four limbs are employed to generate propulsion, characterised by a classic ipsilaterally coordinated tetrapod gait (Table 2, [104]). Though they largely concentrated on Emperor penguins moving and climbing over a variety of substrata, these authors also showed that quadrupedal locomotion was especially effective in smaller penguin species crossing deep, soft snow (see, for example, Chinstrap penguins at https://www.youtube.com/watch?v=zRBHn4REEDY URL last accessed on 7 October 2026).

Wilson et al. [103] addressed the question of why Adélie penguins do not use tobogganing throughout their locomotion over snow, as it is so much more efficient than walking, even uphill. They conducted experiments on anaesthetised and dead penguins that indicated that tobogganing caused disturbance of the plumage and increased friction between feathers and snow. There are problems with these data as the plumages of live, awake penguins are under muscular control, and dead penguins do not secrete preen oil or spread it over their feathers, but their hypothesis that tobogganing carries costs in terms of plumage maintenance and lubrication appears reasonable. In support of this, it should be remembered that penguins have ‘catastrophic moults’. Unlike most flying birds, all feathers are replaced in one period (2–4 weeks, depending on species) during the summer after breeding has finished, and chicks are abandoned [114]. When they return to land/ice to breed, preening and oiling are possible, but replacement of damaged feathers is not. Emperor penguin chicks use tobogganing extensively when travelling from their natal breeding ground to the sea [104], but they do so while they are still in the process of fledging, so that relatively little wear of the first adult plumage will take place.

Several extant temperate penguin species have been seen tobogganing on substrata other than snow or ice. King penguins (*A. patagonicus*) in S. Georgia (see https://www.youtube.com/watch?v=le85xjZPmFs URL last accessed on 7 October 2026) often exhibit tobogganing on sandy beaches as they exit the sea (as well as tobogganing over soft snow; see https://www.youtube.com/watch?v=gvJ2rdShmMM URL last accessed on 7 October 2026). Similarly, Galapágos penguins (*S. mendiculus*) and African penguins (*S. demersus*) toboggan over slippery rocks, well-packed wet sand, or surf edges. Entering or leaving sea water is dangerous for all penguin species, as they are particularly vulnerable to predation (mainly by seals, sharks, and orcas) as they switch from aquatic to terrestrial locomotion. Use of tobogganing rather than walking is more rapid when transiting between media. For example, Emperor penguins jump out of water onto ice by extremely rapid swimming [115] and toboggan for several metres before walking. They also travel at high speed by tobogganing as they reach the ice edge to return to the sea (see https://www.youtube.com/watch?v=snAvGxz7D04 URL last accessed on 7 October 2026). It therefore seems probable that tobogganing was originally part of a water’s-edge escape/transition response but has since become an efficient long-distance mode of locomotion as penguins have spread to cold Subantarctic and Antarctic areas.

#### 3.2.2. Locomotor Evolution

**Figure 17:**
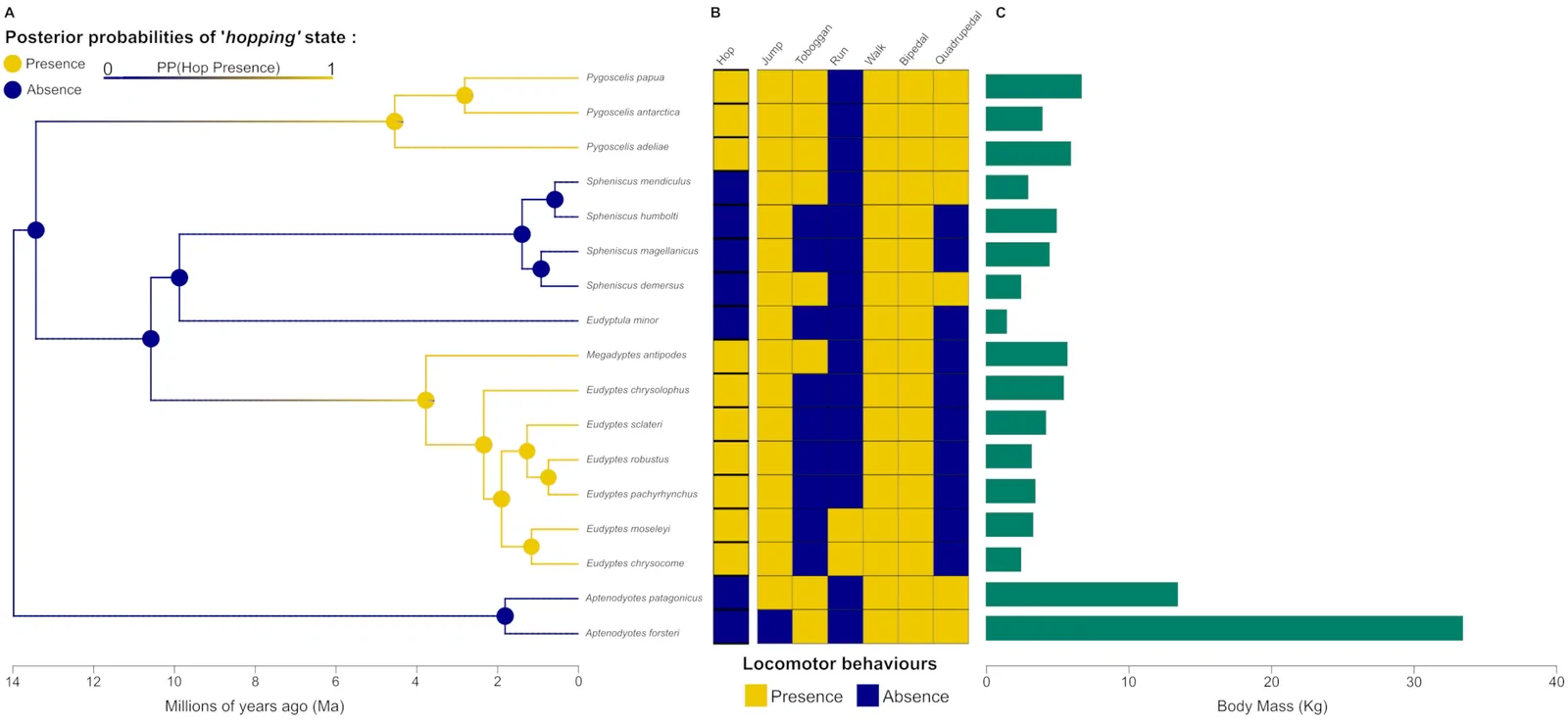
Phylogenetic comparative analysis of penguin locomotor behaviours. (**A**) Ancestral-state reconstruction of hopping behaviour (present/absent) based on the constrained “No hop → Hop” model. Pie charts at each node represent posterior probabilities from stochastic character mapping (*n* = 100), and the branch colour gradient indicates the posterior density of hopping occurrence. (**B**) Behavioural matrix showing the presence or absence of each locomotor pattern (Hop, Jump, Toboggan, Run, Walk, Bipedal, and Quadrupedal) at the tips of the phylogeny. (**C**) Body mass (in kg) for each penguin species.

In penguins (Figure 17), ancestral-state reconstruction favoured the *No-hop → Hop* model, which yielded the lowest AICc (12.22) and BIC (12.78) values. Competing models received slightly higher scores: Equal-Rates (ER) (AICc = 12.86; BIC = 13.43), *Hop → No-hop* (AICc = 13.38; BIC = 13.95), and All-Rates-Different (ARD) (AICc = 14.81; BIC = 15.62), making them less likely given the data.

Under the selected *No-hop → Hop* model, stochastic character mapping (*n* = 100) inferred an average of 2.04 transitions across the phylogeny, consistently pointing toward transitions from non-hopping to hopping species. Furthermore, the character mapping estimated that lineages spent, on average, 59.1% of their evolutionary history in the No hop state, and 40.8% in the Hop state. To assess whether certain locomotor traits may have evolved in a correlated manner, Pagel’s test for discrete character dependence was performed for all pairwise combinations of “*hop*”, “*jump*”, “*toboggan*”, “*run*”, “*walking”*, “*bipedal*”, and “*quadrupedal*” (Figure 17A). In all cases, models assuming independent evolution between locomotor traits were favoured, with no significant associations detected (Supplementary Material File S4), except for two behavioural combinations. First, *hopping* and *quadrupedal* locomotion have evolved dependently and inversely, such that the presence of one generally excludes the other (AIC_dependent_ = 38.34; AIC_independent_ = 39.53; *p* = 0.05), except for the genus *Pygoscelis* (Figure 17B). Second, tobogganing and quadrupedal locomotion also show evidence of congruent evolution, with both behaviours typically co-occurring (AIC_dependent_ = 44.25; AIC_independent_ = 52.06; *p* = 0.003; Figure 17B).

To explore whether body mass could influence the presence or absence of locomotor behaviours, we first assessed the phylogenetic signal of mean mass across penguin species. A moderate but significant phylogenetic signal was detected (K = 0.63, *p* = 0.042; Figure 17 C), suggesting that mass is only partly constrained by phylogeny. This apparent signal may be largely driven by the two heaviest species, both belonging to the genus *Aptenodytes*, which clearly stand out in terms of body mass. We then tested for differences in body mass between species expressing or lacking a given locomotor mode, using either a phylogenetically controlled method (*phyloglm*) or non-phylogenetic Mann–Whitney tests (Supplementary Material File S4). Due to the low number of absences for certain behaviours, jumping, bipedal, and walking locomotor modes could not be statistically tested. No significant differences in body mass were found for any of the other locomotor modes (Supplementary Material File S5). However, although body mass does not appear to influence the expression of the hopping behaviour, this locomotor mode seems to be phylogenetically structured (*phyloglm*: α = 0.02; *p* = 0.85).

### 3.3. Pinnipeds

Pinnipeds are highly aquatic carnivorous mammals. Their fossil history dates to the late Oligocene (29–23 Ma) in the Northern Hemisphere. They comprise three extant families: Phocidae (true seals), Otariidae (fur seals and sea lions), and Odobenidae (walruses). Their evolutionary origins remain debated. Whether pinnipeds evolved from ursid- or mustelid-like ancestors has long been controversial, although molecular evidence indicates that they are equally related to both groups [116]. Likewise, the monophyly of Pinnipedia is now widely accepted based on molecular and combined phylogenetic analyses, whereas earlier morphological studies proposed alternative evolutionary scenarios [29,117]. The phylogenetic position of the walrus (*Odobenus rosmarus*) also remains uncertain, having been proposed as the sister lineage of either Otariidae or Phocidae depending on the analytical framework. Furthermore, comparative analyses of vertebral morphology emphasise functional divergence among the three pinniped families. Phocids possess relatively rigid thoracic regions associated with highly flexible lumbar regions, whereas otariids exhibit highly flexible intervertebral joints throughout the vertebral column, facilitating greater terrestrial and aquatic manoeuvrability. Odobenids display an intermediate condition that more closely resembles phocids [118].

Conventionally, pinniped swimming mechanisms have been divided into otariid forelimb-powered swimmers (underwater fliers) and phocid hindlimb-powered swimmers, with odobenid walruses exhibiting elements of both types of swimming. Otariid forelimbs are specialised flippers, whereas phocid forelimbs retain claws and are more paw-like [119]. However, a recent investigation by Hocking et al. [120] demonstrates that there is a continuum among otariid and phocid pinnipeds, with some true seals of the subfamily Monachinae (e.g., monk seals and leopard seals) exhibiting powerful foreflipper-based swimming. They hypothesise that all pinnipeds had hindlimb-powered ancestors.

Although specialised for efficient swimming, all pinnipeds must return to land or ice to breed. Females give birth to pups that initially have limited blubber reserves and rely on maternal milk and, depending on the species, their natal pelage for insulation during early development. Pups grow rapidly while suckling because pinniped milk is rich in lipids, reaching up to 60% in Weddell seals [119,121]. Adult males also return to terrestrial habitats or nearby coastal waters during the breeding season. In all pinnipeds, delayed implantation results in an approximately annual reproductive cycle, although walruses have a longer reproductive cycle, with a total gestation period (including delayed implantation) of 15–16 months [122]. In addition to reproduction, both sexes and all age classes regularly haul themselves out onto land or ice to moult and rest.

#### 3.3.1. Moving on Land

Phocids generally have more limited terrestrial locomotor abilities than otariids, both in terms of distance and speed [54,123,124,125,126,127,128,129]. Their hindlimbs cannot be rotated forwards beneath the body to support and elevate it above the substratum (Supplementary Material File S6). Consequently, phocids generally travel relatively short distances over land, snow, or ice. By contrast, otariids can support the body on all four limbs and employ quadrupedal walking as well as faster bounding or galloping gaits [126,127,130,131]. They can therefore travel considerable distances from water. One of us (JD) observed large numbers of young male Antarctic fur seals (*Arctocephalus gazella*) at an altitude of 250 m on Signy Island, South Orkneys, in 1987. They had climbed to this elevation over a minimum distance of approximately 1.3 km, demonstrating the ability of otariids to travel considerable distances over steep gradients.

Several modes of progression in phocids differ in the relative contributions of dorsoventral spinal flexion, lateral body undulation, and foreflipper action [54,123,124,125,126,127,128,129,130,131,132,133,134]. A widespread mode combines dorsoventral undulation of the vertebral column with synchronous or nearly synchronous use of the foreflippers [54,132]. However, this mechanism should not be described as true bipedalism because the sternum, ventral body surface, and pelvic region also act as major points of support. The shoulder joints and short humeri of phocids lie largely within the body contour, with the externally visible foreflippers emerging near the elbow [127]. The hindlimbs are highly modified for aquatic propulsion and cannot be rotated beneath the body to support terrestrial locomotion [130,132]. This condition is particularly pronounced in elephant seals, in which the paired hindflippers form a functionally symmetrical propulsive surface and are dragged passively during terrestrial progression [133,134].

**Figure 18:**
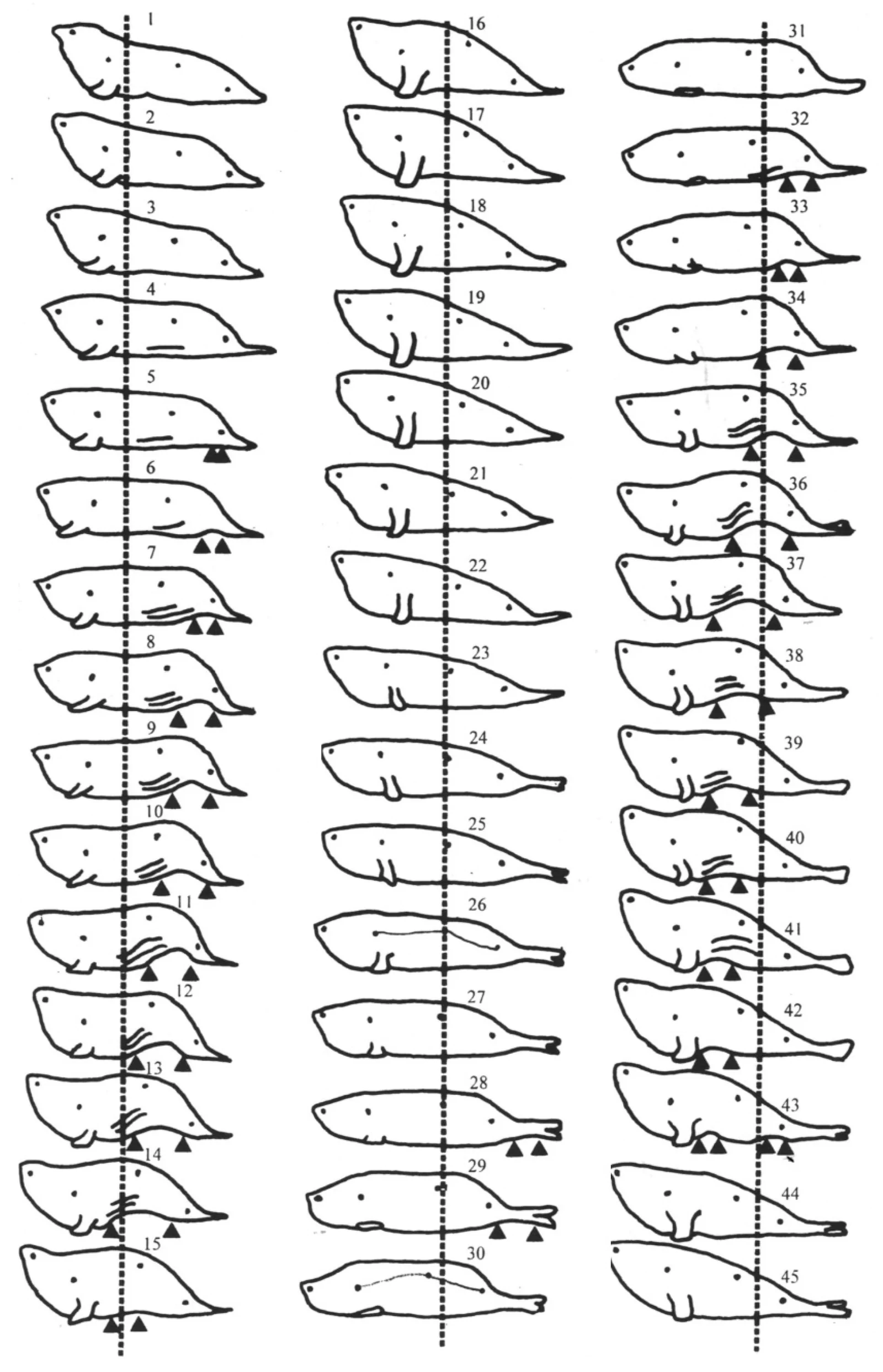
Caterpillar-like movement of an adult male southern elephant seal (Mirounga leonina) filmed at 25 frames s^−1^ from a distance of approximately 10 m at Karakkatta Bay, South Georgia, in 1993 (Davenport and Bels, unpublished data). The seal was filmed from the side as it moved rapidly over flat tussock grass. Three spots of non-toxic whitewash were applied to its left side: one above the forelimb, one approximately midway between the forelimb and hindlimb, and one near the hindflipper. Numerals indicate frame numbers over several locomotor cycles. Vertical dashed lines indicate a fixed point passed by the seal. Pairs of black triangles delimit portions of the ventral body surface raised above the substratum.

The caterpillar-like mode of phocid locomotion involves alternating support by the pelvic and sternal regions. At the beginning of a locomotor cycle, flexion of the lumbar region raises and advances the pelvis while most of the anterior body weight is supported by the sternum. The dorsoventral wave produced by spinal flexion then travels anteriorly. As the pelvic region returns to the substratum, the thoracic and abdominal regions rise and the foreflippers support the anterior part of the body. Extension and posterolateral movement of the foreflippers assist the forward transfer of the sternum, head, and neck. The foreflippers are subsequently lifted and repositioned anteriorly while renewed lumbar flexion initiates the next cycle. Throughout this sequence, the hindflippers are dragged passively and make no active contribution to propulsion [54,132]. Repetition of the cycle produces forward progression over the substratum, as illustrated by the southern elephant seal in Figure 18.

Considerable variation nevertheless occurs among phocids (Table 3). In the adult Weddell seal *Leptonychotes weddellii*, terrestrial progression is normally produced by caterpillar-like dorsoventral undulation without active use of the foreflippers [128]. The sternum is therefore not raised by foreflipper extension, and the ventral surface slides over the ice or snow. Inspection of recent film (Website 3, Supplementary Material File S1C) reveals an additional feature of Weddell seal locomotion. In thick snow, the head is moved from side to side to clear a path before extension of the back pushes the anterior region forwards.

Ross, crabeater, and leopard seals can also progress without appreciable foreflipper involvement, although crabeater and leopard seals additionally employ a faster sinuous mode [128]. In this mode, lateral undulation of the body is combined with alternating action of the foreflippers. O’Gorman [54] described this “swimming-like” progression as particularly well developed in the crabeater seal *Lobodon carcinophaga*, which can maintain it over considerable distances. Interaction between the hindflippers and the substratum may occur, although inspection of recent videos (Websites 5 and 6, Supplementary Material File S1C) indicates that the interdigital webs of the hindflippers are not fully expanded during movement over snow or ice. Leopard seals can also employ this mode, generally during shorter locomotor sequences [128].

Elephant seals normally progress through dorsoventral spinal undulation combined with synchronous foreflipper support, while the hindflippers are dragged passively [131]. However, some large male southern elephant seals (*Mirounga leonina*) have been observed moving down steep gravel beaches without the foreflippers contacting the substratum (personal observation, JD). Adult male southern elephant seals can exceed a body mass of 3.5 tonnes and are substantially larger than females. Their terrestrial locomotion is therefore constrained not only by the aquatic specialisation of the limbs but also by their exceptionally large body mass.

Large beachmaster males also adopt threat and fighting postures involving limited or absent limb support [133]. They commonly raise the head and neck beyond the vertical, causing the foreflippers to leave the substratum before delivering strikes towards an opponent’s head or neck. Alternatively, a male may elevate and curve the posterior part of the body so that the extended hindflippers are held high above the substratum while the head is thrown backwards. This produces a bow-shaped posture, concave dorsally, from which the animal can rock forwards and strike without active foreflipper propulsion.

Arctic and subarctic regions are inhabited by several northern ice seals of the subfamily Phocinae, including harp, bearded, hooded, and ringed seals. These species generally have forwardly directed forelimbs with strong claws and relatively mobile wrists [130,135]. Their terrestrial locomotion is difficult to study under natural conditions, and many published descriptions are based on animals induced to move rapidly. Recent films of harp seals *Pagophilus groenlandicus* (Websites 8 and 9, Supplementary Material File S1C) show that the hindflippers are held above the substratum while propulsion is generated by alternate action of the forelimbs. The foreflippers function as clawed paws that grip the snow or ice and pull the body forwards. Lateral sinuous movements and dorsoventral spinal waves accompany this action, but the hindflippers make no direct contribution to propulsion.

**Figure 19:**
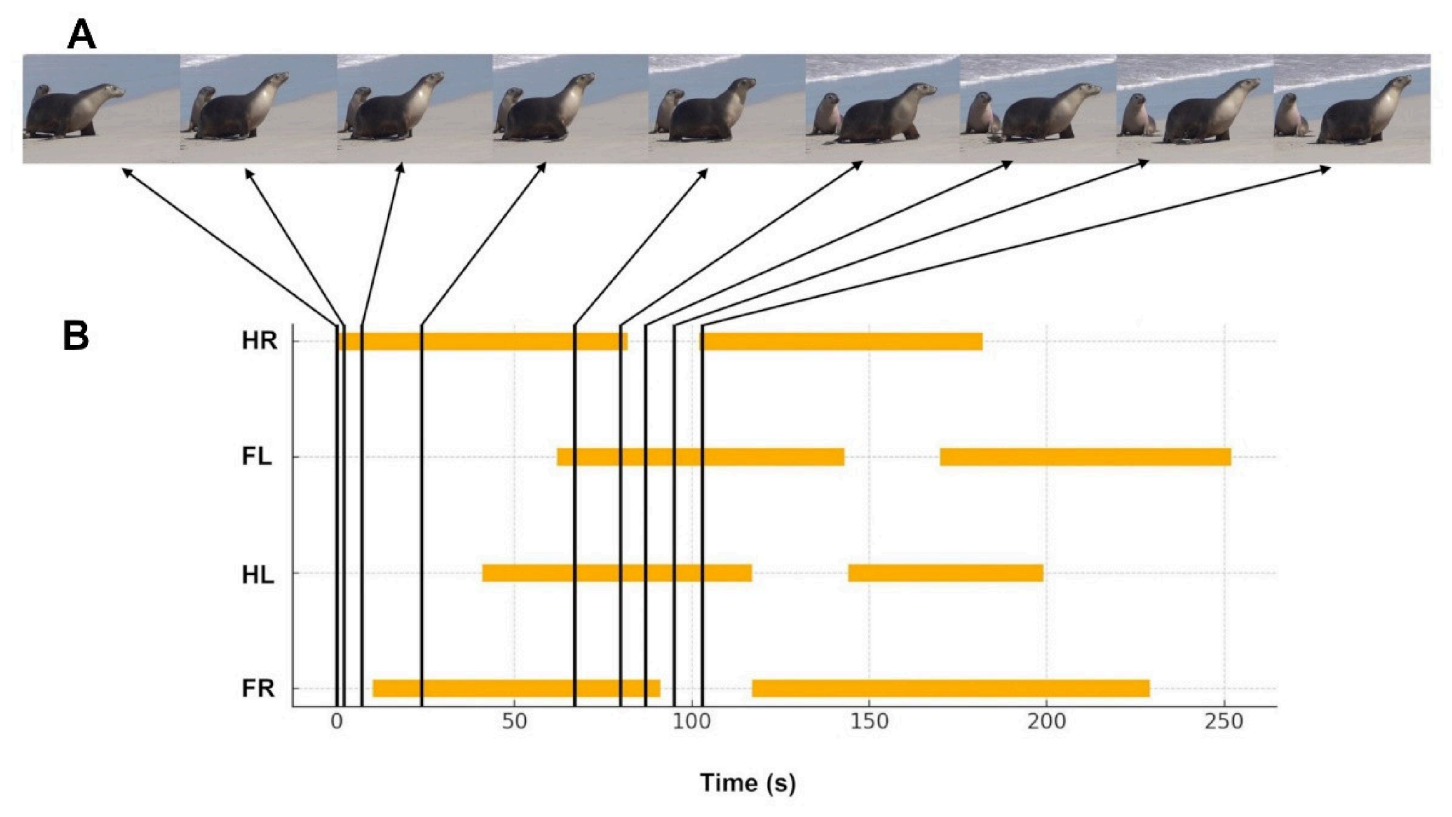
Representative gait diagram of *Neophoca cinerea* moving over sand at Seal Bay Conservation Park, Kangaroo Island, Australia. (**A**) Representative postures during successive forelimb and hindlimb stance and swing phases. (**B**) Two successive locomotor cycles. Yellow bars indicate stance phases, whereas white intervals indicate swing phases. Abbreviations: FL, left forelimb; FR, right forelimb; HL, left hindlimb; HR, right hindlimb. Photo credit: Florence Kirchhoff.

Otariid terrestrial locomotion (Figure 19) has been investigated, particularly in the California sea lion *Zalophus californianus* [126,127,131]. During walking, the limbs are used in an alternating quadrupedal sequence, broadly comparable to the walking patterns of terrestrial tetrapods [126,131]. However, the limited separation of the hindlimbs restricts stride length. Walking is accompanied by lateral and dorsoventral movements of the vertebral column. Recent films show comparable movements in the smaller Galápagos sea lion *Zalophus wollebaeki* (Supplementary Material File S1C).

California sea lions can progress more rapidly by using a bounding or galloping gait [127,131]. During this gait, the foreflippers move almost synchronously, although they remain slightly out of phase, whereas the hindlimbs move together. The heels contact the substratum and function as pivots. Much of the forward impulse is generated by flexion and extension of the vertebral column, powered by the axial musculature [127]. This bounding gait can be used at relatively low speeds and is mechanically and energetically more effective than the undulatory terrestrial progression of phocids [54,127,132]. Similar bounding movements can be observed in the Steller sea lion in the films included in Supplementary Material File S1C.

Although this represents the generally accepted description of otariid terrestrial locomotion, interspecific differences occur in the relative contributions of the axial skeleton and limbs [126,134]. Beentjes [132], comparing the Hooker’s sea lion *Phocarctos hookeri* and the New Zealand fur seal *Arctocephalus forsteri*, proposed that sea lions and fur seals differ in their walking mechanisms. In the New Zealand fur seal, the hindlimbs tended to move together while the foreflippers acted alternately, with propulsion relying strongly on flexion of the posterior axial skeleton. Film of the Antarctic fur seal *Arctocephalus gazella* (Website 12) appears consistent with this interpretation. However, recent film of a New Zealand fur seal (Website 13, Supplementary Material File S1C) shows a walking pattern similar to that of Galápagos sea lions. The extent and functional significance of interspecific variation in otariid terrestrial locomotion therefore require further investigation.

Inspection of Website 15 (Supplementary Material File S1C) shows a large walrus leaving the water on a sandy beach using a bipedal mechanism without hindlimb involvement, much like a phocid such as an elephant seal, but with alternate foreflipper action. This appears to be an inefficient locomotor mode because the ventral surface between the foreflippers is not lifted from the substratum. By contrast, a recently published film (BBC 2022, Supplementary Material File S1C) shows that adult walruses have a rapid and efficient method of moving down a steep sandy beach: they roll down the slope with the body axis perpendicular to the direction of travel and the forelimbs held tightly against the body.

Walrus terrestrial locomotion differs from both the predominantly undulatory progression of phocids and the elevated quadrupedal walking of otariids. Gordon [133] has provided the seminal description of walrus (odobenid) terrestrial locomotion. Adult walruses exhibit a lateral-sequence, diagonal-couplets walk (i.e., like other terrestrial tetrapods), with all four limbs involved, but this is combined with a forward lunge, during which the spine is flexed and the hindlimbs are used to drive the bulk of the body and head forwards. The ventral surface of the body is clear of the substratum for only part of the limb cycle, during the forward lunge. This mode of locomotion (see Website 14, Supplementary Material File S1C) therefore contains elements of the phocid and otariid mechanisms described above.

#### 3.3.2. Locomotor Evolution

**Figure 20:**
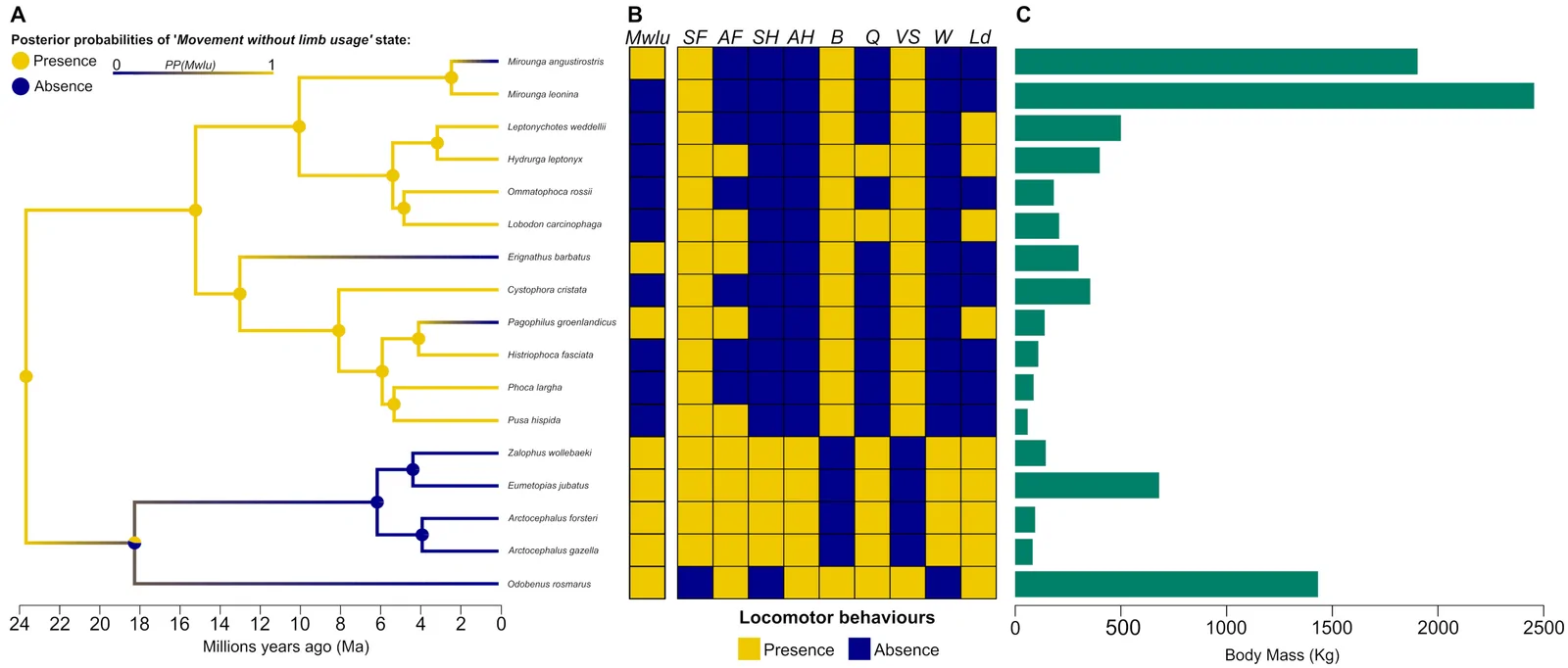
Phylogenetic comparative analysis of pinniped locomotor behaviours. (**A**) Ancestral-state reconstruction of limb use (present/absent), based on the ‘No limb use → Limb use’ model. Pie charts at each node represent posterior probabilities from stochastic character mapping (*n* = 100), and the branch colour gradient indicates the posterior probability of limb use along the branches. (**B**) Behavioural matrix showing the presence or absence of each locomotor pattern at the tips of the phylogeny: Lu (limb use), SF (synchronous forelimb movement), AF (asynchronous forelimb movement), SH (synchronous hindlimb movement), AH (asynchronous hindlimb movement), B (bipedal locomotion), Q (quadrupedal locomotion), VS (ventral-surface locomotion), W (walking), and Ld (lateral undulation). (**C**) Body mass (kg) for each pinniped species.

Ancestral-state reconstruction of limb use (Figure 20A) identified the directional model assuming transitions from absence to presence of limb use as the best-fitting model (AICc = 22.63; BIC = 23.19). This model received greater support than the All-Rates-Different model (ARD; AICc = 25.19; BIC = 25.99) and the Equal-Rates model (ER; AICc = 25.56; BIC = 26.40), although the differences among these three models were modest. In contrast, the reverse directional model (presence → absence) showed a considerably poorer fit (AICc = 32.64; BIC = 33.20). The absence → presence model was therefore retained for stochastic character mapping. Under this model, stochastic character mapping (*n* = 100) revealed an average of 4.45 state transitions across the phylogeny. On average, lineages spent 63.79% of their evolutionary history in the ‘absence of limb use’ state and 36.20% in the ‘presence of limb use’ state.

To assess whether certain locomotor behaviours have evolved in a correlated manner, we performed Pagel’s test for discrete trait dependence (Figure 20B). Among all combinations tested, only three trait pairs showed statistically significant evidence of dependent evolution. All three associations were congruent, meaning that the two behaviours tended to co-occur. These included (i) limb use and asynchronous forelimb movement (AIC_(dependent) = 48.68; AIC_(independent) = 51.48; *p* = 0.03); (ii) asynchronous forelimb use and quadrupedal locomotion (AIC_(dependent) = 45.77; AIC_(independent) = 49.31; *p* = 0.02); and (iii) quadrupedal locomotion and lateral undulation (AIC_(dependent) = 41.82; AIC_(independent) = 47.08; *p* = 0.01). Eight other trait combinations did not reach statistical significance (*p* = 0.06 in each case) and were therefore not interpreted as evidence of dependent evolution.

We tested whether body mass exhibited a phylogenetic signal in pinnipeds and detected a significant signal (Blomberg’s K = 1.01, *p* = 0.004), indicating that closely related species tend to have similar body masses. To assess whether body mass influenced the expression of specific locomotor behaviours, we conducted phylogenetically controlled logistic regressions (*phyloglm*). None of these models revealed a significant relationship between body mass and the presence or absence of any locomotor behaviour (Supplementary Material File S8). However, several behaviours appeared to exhibit strong phylogenetic structuring, with closely related species tending to share the same locomotor traits. This pattern was observed for limb use, synchronous forelimb movement, asynchronous hindlimb movement, bipedal locomotion, ventral-surface locomotion, walking, and synchronous hindlimb movement. To test the relationships between body mass and locomotor behaviours without phylogenetic correction, we also performed Mann–Whitney U tests. These tests likewise revealed no significant differences in body mass between behaviourally defined groups (Supplementary Material File S7).

## 4. Discussion

### 4.1. Adapted Marine Tetrapods Moving on Land: Evolution of the Locomotor Repertoires

Locomotor behaviour in tetrapods has been profoundly modified during the secondary return of several lineages to aquatic habitats, although the extent of these changes varies considerably among clades [135,136,137]. Cetaceans and extant sirenians have completely lost the ability to move on land, as the progressive specialisation of their locomotor systems for efficient aquatic propulsion has effectively precluded terrestrial locomotion. In contrast, representatives of the three major lineages considered in this review have retained terrestrial locomotor abilities despite exhibiting striking convergent modifications of the fore- and hindlimbs. These transformations are generally interpreted as resulting from a combination of adaptive evolution, phylogenetic constraints, and developmental processes [13]. Although these changes are readily apparent at the morphological level, including limb structure and articulation, body shape, body-surface characteristics, and body mass (Table 1, Table 2 and Table 3), they are also likely to involve modifications of (i) musculoskeletal anatomy and physiology [135,136,137], (ii) biomechanical function and the evolutionary diversification of locomotor systems [135,136,137], (iii) the integration of visual, auditory, proprioceptive, and vestibular sensory information [138,139,140], and (iv) the neural mechanisms underlying locomotor control, including central pattern generators, postural coordination, and the generation of rhythmic behaviour [141,142,143,144,145,146,147,148,149,150]. Together, these components contribute to locomotor performance in aquatic and terrestrial environments.

**Figure 21:**
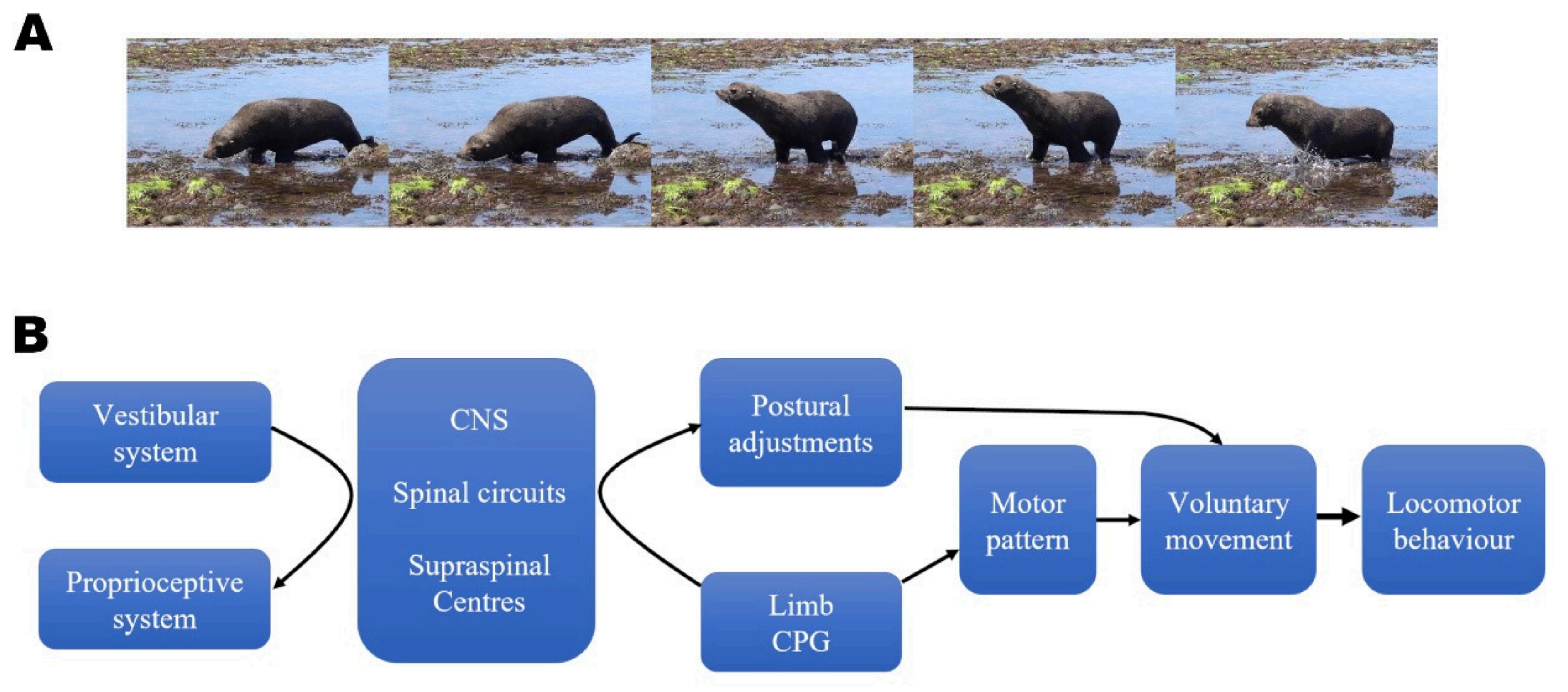
(**A**) Representative sequence illustrating synchronised forelimb and hindlimb movements during rapid terrestrial locomotion in the Australian sea lion, *Neophoca cinerea*. The sequence highlights the associated postural adjustments of the body throughout a locomotor cycle. Photo credit: Florence Kirchhoff. (**B**) Conceptual summary of the sensorimotor organisation underlying terrestrial locomotion in secondarily aquatic marine tetrapods, adapted from general tetrapod locomotor circuitry. The model illustrates the integration of vestibular and proprioceptive sensory inputs with supraspinal centres, spinal circuits, and limb central pattern generators (CPGs), resulting in postural adjustments, motor pattern generation, voluntary movement, and the expression of terrestrial locomotor behaviour.

Although the neural mechanisms underlying locomotor control remain poorly understood in secondarily aquatic tetrapods, the diversity of terrestrial locomotor repertoires described here suggests that similar morphological adaptations for aquatic propulsion have been accompanied by different motor strategies on land [54,84,110,132,145,146,147,148,149]. Future studies integrating behavioural analyses with biomechanics and neurophysiology will be necessary to determine how locomotor control has evolved during repeated transitions between terrestrial and aquatic environments (Figure 21). The marked differences observed among marine turtles, penguins, and pinnipeds also suggest that the effects of postural control on terrestrial locomotor ability vary according to body organisation and possibly to the locomotor repertoire used in water [54,84,110,126,132,144,146,147,148,149,150]. Quantitative studies combining three-dimensional kinematics, biomechanics, and musculoskeletal analyses should clarify how postural adjustments contribute to terrestrial locomotor performance and to the evolutionary diversification of terrestrial locomotion.

### 4.2. Evolution of Swimming and Terrestrial Locomotion

Marine tetrapods exhibit remarkable diversity in swimming strategies, ranging from body undulation to appendicular propulsion or combinations of both, and reflecting repeated evolutionary solutions to aquatic locomotion [4,5,6,9,144,145,146,147,148,149,150]. Despite this diversity, all extant representatives of the three lineages considered in this review retain some degree of terrestrial locomotor ability that remains essential during key life-history stages, including reproduction, moulting, and resting. This functional duality raises important questions concerning the evolution of terrestrial locomotor repertoires and the extent to which these behaviours have been constrained or diversified during repeated transitions between terrestrial and aquatic environments.

Current evidence suggests that fundamental principles of the neural organisation of locomotion may be more evolutionarily conserved than the musculoskeletal structures that generate movement [140,141,142]. Accordingly, repeated transitions to aquatic life may have involved profound modifications of morphology and biomechanics while retaining basic principles of locomotor organisation. However, the extent to which locomotor control has been conserved, modified, or independently reorganised in secondarily aquatic tetrapods remains largely unresolved and represents an important avenue for future research.

Within this framework, our comparative analyses indicate that distinct terrestrial locomotor repertoires have evolved despite profound specialisation for aquatic propulsion. Rather than supporting a single convergent evolutionary pathway, our results are consistent with multiple functional solutions linking morphology, biomechanics, and behaviour. In the following sections, we compare the principal biomechanical similarities and differences between aquatic and terrestrial locomotion in sea turtles, penguins, and pinnipeds before discussing the evolutionary implications of our comparative analyses.

#### 4.2.1. Sea Turtles

Marine turtles exhibit the most conservative terrestrial locomotor repertoire among the extant marine tetrapods considered here. Despite marked differences in body size and ecology, all extant species retain a broadly similar terrestrial locomotor organisation based primarily on coordinated movements of the fore- and hindlimbs [17,38,81,82,83,84]. This remarkable conservatism is probably explained by the rigid shell, which encloses both the pectoral and pelvic girdles and greatly restricts axial movements of the trunk [17,38,84]. Consequently, terrestrial propulsion relies almost entirely on coordinated limb actions, whereas trunk movements contribute little to body progression compared with penguins and pinnipeds. These biomechanical constraints probably limit the range of terrestrial locomotor strategies that can evolve in marine turtles.

**Figure 22:**
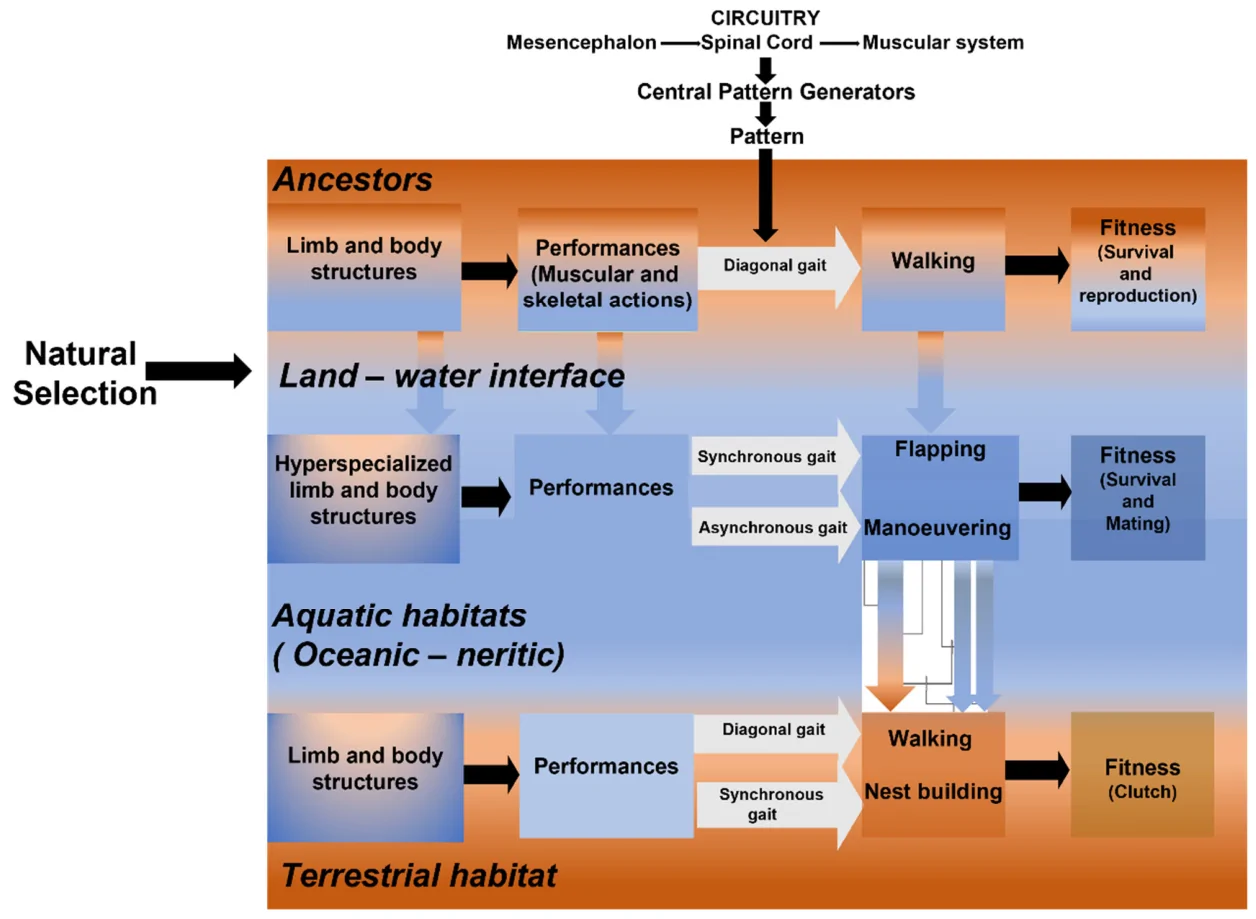
Conceptual framework illustrating the relationships among locomotor circuitry, morphology, biomechanical performance, locomotor behaviour, and fitness during the secondary transition from terrestrial to aquatic environments in marine turtles. Natural selection may favour modifications of limb and body morphology that alter locomotor performance and behaviour, while fundamental components of the neural locomotor circuitry inherited from terrestrial tetrapods may be retained. Progressive specialisation of the appendicular system for aquatic propulsion leads to changes in biomechanical performance and locomotor behaviour in aquatic environments, whereas terrestrial locomotion is retained for reproduction through modified walking patterns and nest-building behaviour.

Although hatchlings and nesting females experience markedly different biomechanical constraints, both life stages share the same fundamental locomotor organisation [15,17,81,82,83,84]. Marine turtles therefore provide a useful model for examining how terrestrial locomotor repertoires have been maintained despite profound specialisation for aquatic propulsion (Figure 22). Swimming is characterised by synchronous, lift-based foreflipper propulsion, in which thrust is generated through cyclic downstroke and recovery phases [4,14,17,84]. In contrast, terrestrial locomotion relies on both synchronous and asynchronous limb coordination, depending on species, developmental stage, and behavioural context [15,82,83,84,85].

Comparison of swimming and terrestrial kinematics reveals both conserved and modified components of the locomotor cycle. During swimming, propulsion is generated during the downstroke, whereas the upstroke and recovery phases reposition the foreflipper for the next propulsive cycle [4,14,17,84]. During terrestrial locomotion, the stance phase similarly generates forward propulsion, whereas the swing phase repositions the foreflipper through protraction and axial rotation before the next contact with the substratum [15,82,83,84]. Although foreflipper trajectories differ markedly between water and land, the alternation of propulsive and recovery phases is maintained, suggesting the functional reorganisation of a shared underlying locomotor architecture rather than the evolution of an entirely novel locomotor repertoire. Hindlimb function also differs substantially between the two environments, contributing primarily to stabilisation and manoeuvring during swimming [14,17,84], but to body support and propulsion during terrestrial locomotion [15,82,83,84].

Within this biomechanical framework, our comparative analyses suggest that asynchronous terrestrial locomotion most probably represents the ancestral condition in marine turtles, whereas synchronous locomotion evolved independently in a limited number of lineages. Hatchlings of most cheloniids retain both locomotor patterns and can switch between them according to behavioural context; for example, during nest emergence or when negotiating irregular substrata [17,82,84]. In contrast, *Dermochelys coriacea* relies exclusively on synchronous terrestrial locomotion throughout its life cycle [15,83,84]. These observations indicate that different terrestrial locomotor repertoires evolved while maintaining the common functional requirement of efficient progression across sandy substrata.

The repeated occurrence of synchronous terrestrial locomotion in large-bodied species is consistent with the hypothesis that increasing body size and associated biomechanical constraints influenced the evolution of terrestrial gait [15,83,84]. However, our comparative analyses do not demonstrate a direct causal relationship between body mass and locomotor pattern. Instead, they suggest that several interacting factors, including morphology, biomechanics, and ecological specialisation, probably shaped the diversification of terrestrial locomotor repertoires. Fossil evidence and comparative analyses document transformations of the appendicular skeleton associated with transitions to aquatic and marine life [38,73,84], but provide only indirect evidence regarding terrestrial locomotor behaviour.

#### 4.2.2. Penguins

Penguins represent a unique example among secondarily aquatic tetrapods in which highly specialised underwater flight coexists with a remarkably diversified terrestrial locomotor repertoire. Swimming propulsion is generated almost exclusively by synchronous, lift-based movements of the forelimbs, whereas the hindlimbs contribute primarily to steering and body stabilisation [90,91,145,146,147,148,149,150]. During terrestrial locomotion, however, propulsion relies almost entirely on the hindlimbs, whereas the forelimbs may contribute only to balance and postural control. This marked functional dissociation between aquatic and terrestrial locomotion is particularly apparent during exit from water. Penguins can transform a nearly horizontal swimming posture into an upright bipedal posture before or at substratum contact, positioning the hindlimbs for immediate terrestrial support and progression. Among the lineages considered here, this rapid postural transition is distinctive because marine turtles and pinnipeds retain a more nearly horizontal body orientation during both emergence and subsequent terrestrial locomotion. Penguins can also use porpoising during rapid surface travel, alternating submerged propulsion with aerial phases above the water surface [144].

**Figure 23:**
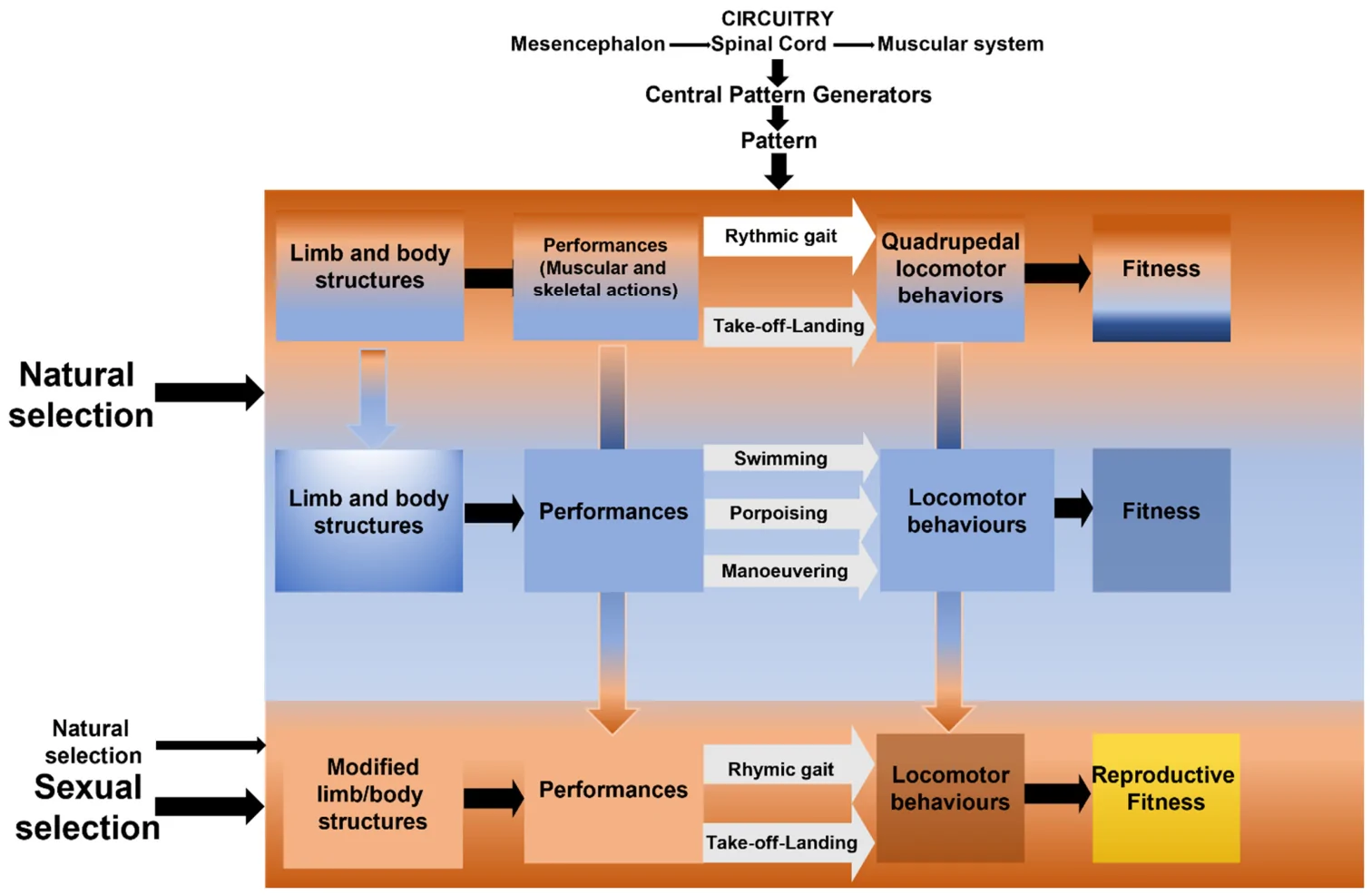
Conceptual framework illustrating the evolutionary relationships among neural locomotor circuitry, morphology, biomechanical performance, locomotor behaviour, and fitness during the secondary transition from terrestrial to aquatic environments in penguins and pinnipeds. Hypothesised conserved components of the neural locomotor circuitry, including central pattern generators (CPGs), provide the basis for rhythmic locomotion in aquatic and terrestrial environments. Progressive morphological specialisation of the appendicular system modifies biomechanical performance and gives rise to distinct aquatic and terrestrial locomotor repertoires. Terrestrial locomotion is nevertheless retained for essential life-history functions, including reproduction, moulting, and resting, as well as for transitions between aquatic and terrestrial environments.

On land, unlike sea turtles, penguins exhibit a highly diversified locomotor repertoire that includes walking, jumping, hopping, and, in several species, tobogganing [103,104] (Figure 23). Comparison of these terrestrial locomotor modes suggests that their evolution primarily involved modifications to hindlimb coordination and body posture rather than fundamental changes in forelimb function. Jumping and hopping require substantial dorsoventral body movements associated with hindlimb extension [112], whereas tobogganing represents an alternative locomotor solution that permits efficient progression across snow and ice while reducing the energetic costs associated with walking [108]. These different locomotor modes illustrate how biomechanical adaptations permit the exploitation of contrasting terrestrial habitats despite extreme specialisation for aquatic locomotion.

Within this framework, our comparative analyses indicate that hopping occurs in a restricted set of penguin lineages. The No hop → Hop model had the lowest AICc and BIC values, but the differences between the competing models were small and therefore provide limited evidence concerning the direction of evolutionary change. Tobogganing and quadrupedal locomotion showed significant evidence of dependent and congruent evolution, consistent with their combined use during progression over snow and ice. Hopping and quadrupedal locomotion showed a weaker inverse association, except in the genus *Pygoscelis*. No significant relationship was detected between body mass and the presence of hopping or the other locomotor behaviours that could be tested. These results do not support body mass as the primary determinant of terrestrial locomotor diversity in penguins, and the possible influence of habitat characteristics remains to be directly tested. The fossil record provides a complementary morphological perspective: some penguins show variation in tarsometatarsal proportions and hindlimb robustness [105,110], indicating that hindlimb morphology was not uniform during penguin evolution. This variation is compatible with differences in terrestrial locomotor capabilities, but specific locomotor behaviours cannot be reconstructed confidently from osteology alone.

#### 4.2.3. Pinnipeds

Pinnipeds illustrate contrasting evolutionary solutions to aquatic locomotion (Figure 23). Phocids primarily generate propulsion through lateral movements of the trunk and hindflippers, whereas otariids rely on synchronous, lift-based foreflipper propulsion, with the hindlimbs contributing mainly to steering and manoeuvring [7,25,118,120]. Walruses combine elements of both swimming patterns, relying predominantly on hindlimb propulsion while the forelimbs contribute mainly to manoeuvring [130,134]. Despite these marked differences during swimming, all three groups retain terrestrial locomotor abilities, although they employ fundamentally different biomechanical strategies. Phocids combine trunk undulation with variable forelimb use, otariids rely predominantly on quadrupedal limb support, and walruses can employ both quadrupedal and forelimb-dominated progression [54,126,132,133,134]. These contrasting locomotor patterns demonstrate that similar ecological requirements for moving on land have been met through different biomechanical solutions.

Comparison of aquatic and terrestrial locomotion further highlights how locomotor functions have been reorganised during transitions between environments. In phocids, the hindflippers provide the main propulsive force during swimming [148,149] but contribute little or not at all during terrestrial locomotion, where propulsion depends primarily on trunk movements and, in many species, synchronous or asynchronous forelimb actions [54,128,132]. Conversely, otariids use the foreflippers as the principal propulsive organs both in water and during terrestrial progression, although their mechanical role differs markedly between lift generation during swimming and weight support and propulsion during walking [25,126,131,134,150]. Walruses combine these functional patterns, using all four limbs during typical terrestrial progression while retaining the ability to move using the forelimbs without hindlimb involvement. These observations indicate that the same anatomical structures have been functionally reorganised to meet the distinct mechanical demands imposed by aquatic and terrestrial locomotion.

Within this biomechanical framework, ancestral-state reconstruction identified the absence → presence of limb-use model as the best-fitting model, although its advantage over the ARD and ER models was modest. The direction of evolutionary change in limb use should therefore be interpreted cautiously. The comparative data nevertheless demonstrate marked differences among extant pinniped lineages. Phocids exhibit the greatest diversity, combining body undulation with no limb use or with synchronous or asynchronous forelimb actions; because the hindlimbs generally do not contribute to propulsion on land, the latter patterns are classified here as functionally bipedal, although the sternum, abdomen, and pelvic region may also provide body support. Otariids consistently rely on quadrupedal locomotion and show no evidence of ventral sliding in the present dataset, whereas walruses combine quadrupedal progression with forelimb-dominated locomotion. No significant relationship was detected between body mass and the presence or absence of the locomotor behaviours examined. Body mass alone therefore does not account for the diversification of terrestrial locomotion in pinnipeds, although the contributions of lineage-specific morphology, biomechanics, and ecology remain to be tested. Overall, the diversification of terrestrial locomotor repertoires in pinnipeds appears to have resulted from different functional reorganisations of a common mammalian locomotor system rather than from a single evolutionary trajectory. However, the relative contributions of morphology, biomechanics, and neural control remain unresolved.

More generally, comparisons between marine turtles, penguins, and pinnipeds reveal that specialisation for aquatic propulsion has not produced convergence towards a single terrestrial locomotor repertoire. Marine turtles retain a predominantly appendicular system constrained by the shell and characterised by a relatively limited range of terrestrial coordination patterns. Penguins exhibit a pronounced functional separation between forelimb-powered swimming and hindlimb-powered terrestrial locomotion, allowing the expression of walking, jumping, hopping, tobogganing, and occasional quadrupedal progression. Pinnipeds show the greatest diversity in the contributions of axial undulation, forelimb action, hindlimb function, and ventral body support. Across the three lineages, terrestrial locomotor diversity therefore cannot be attributed to body mass, limb morphology, or aquatic specialisation considered independently. It emerges from the integration of inherited body organisation, musculoskeletal properties, biomechanical constraints, sensorimotor control, substrate characteristics, and the ecological functions for which animals return to land or ice. From this perspective, locomotor repertoires should be analysed not simply as collections of discrete gaits, but as organised behavioural sequences in which posture, axial movements, limb coordination, and environmental interactions change through time [151,152]. Combining this sequence-based approach with ecomechanical models and functional-trait analyses should provide a more comprehensive understanding of how phenotypic diversity arises during repeated evolutionary transitions between terrestrial and aquatic environments.

## 5. Limitations and Future Directions

Behavioural characters were coded at the species level, with each species contributing a single state irrespective of the number of publications, videos, or original observations used to establish it. Effective sample sizes and character-state distributions are therefore constrained by the extant diversity of each lineage and by the observed taxonomic distribution of the behaviours, and not by the volume of available documentation or by an incomplete experimental sampling design. States restricted to few species inevitably reduce the precision and statistical power of ancestral-state reconstructions and correlated-evolution analyses, thereby increasing the risk of failing to detect genuine evolutionary associations (Type II error). Sparse and uneven character-state distributions, together with the examination of multiple character pairs and competing evolutionary models, may make isolated significant associations or model preferences unstable. Accordingly, results based on nominal significance or small differences in AICc and BIC values are treated as exploratory comparative evidence rather than definitive evolutionary trajectories. Because the number of extant species cannot be increased, the robustness of these inferences must instead be evaluated through additional independent behavioural observations, alternative phylogenetic hypotheses, sensitivity analyses, and comparison among evolutionary models. The resulting conclusions remain conditional on current phylogenies, behavioural coding, and evolutionary models, and may also be refined by future palaeontological evidence. Nevertheless, the analyses distinguish clearly different terrestrial locomotor repertoires associated with independent transitions to aquatic life and convergent specialisation for swimming.

Future studies should combine standardised behavioural observations with quantitative analyses of external morphology, internal skeletal architecture, joint organisation, muscle anatomy, and whole-body biomechanics. Modern three-dimensional imaging, including post-mortem computed tomography, micro-CT, and virtual reconstruction, together with biomechanical modelling, should help determine how structural modifications associated with aquatic specialisation constrain or facilitate locomotion on land. Because marine turtles, penguins, and pinnipeds represent three independent transitions between terrestrial and aquatic environments, their comparison provides an exceptional opportunity to link structural evolution, locomotor performance, behavioural repertoires, and fitness [6,140,151,152].

## 6. Conclusions

(1) The independent transitions of sea turtles, penguins, and pinnipeds from terrestrial to marine environments provide a powerful comparative framework for understanding how locomotor systems are organised for moving in water and on land.

(2) Each lineage has evolved a distinct functional solution. Marine turtles retain the most conservative terrestrial repertoire, constrained largely by the shell and dominated by coordinated limb movements. Penguins combine forelimb-powered underwater flight with hindlimb-powered walking, jumping, hopping, and, in some species, tobogganing and quadrupedal progression. Pinnipeds exhibit the greatest diversity in axial movements, limb use, and interlimb coordination during terrestrial locomotion.

(3) Comparative and phylogenetic analyses show that terrestrial locomotor diversity cannot be explained by body mass or aquatic specialisation alone. Instead, it emerges from interactions among inherited body organisation, biomechanical constraints, evolutionary history, sensorimotor control, and the ecological and substrate conditions encountered on land or ice.

(4) Adaptation to efficient swimming does not, therefore, impose a single evolutionary pathway on terrestrial locomotion. Similar aquatic demands can coexist with markedly different terrestrial repertoires because ancestral locomotor functions can be retained, restricted or reorganised in lineage-specific ways.

(5) This study provides the first integrative comparison of terrestrial locomotor repertoires across three extant marine tetrapod lineages that retain terrestrial locomotion despite extensive transformation of their limbs into flippers. The resulting behavioural and evolutionary framework establishes a foundation for future studies linking locomotor sequences, morphology, neural control, biomechanical performance, ecology, and fitness during repeated transitions between land and water.

## Figures and Tables

**Table 1:** Comparison of approximate body masses and forelimb movements during terrestrial locomotion in adult and hatchling marine turtles. P indicates that synchronous forelimb movements were present; A indicates that locomotion was based on asynchronous forelimb movements.

Species	Common Name	Adult Mean Body Mass (kg)	Adults	Hatchlings	References
*Dermochelys coriacea*	Leatherback turtle	475.0	P	P	[41]
*Caretta caretta*	Loggerhead turtle	110.0	A	A	[41,42]
*Chelonia mydas*	Green turtle	190.0	P	A	[41,43]
*Eretmochelys imbricata*	Hawksbill turtle	80.0	A	A	[41,44]
*Lepidochelys kempii*	Kemp’s ridley turtle	40.0	A	A	[41,45]
*Lepidochelys olivacea*	Olive ridley turtle	62.0	A	A	[41,46]
*Natator depressus*	Flatback turtle	125.0	P	A	[41,47]

**Table 2:** Comparison of body masses and modes of terrestrial locomotion in adult penguins. P indicates that a mode was present in a species; A indicates that a mode was absent. Adult body masses correspond to mean values calculated from published body-mass ranges.

Species	Common Name	Adult Mass Range (kg)	Adult Mean Mass (kg)	Hop	Jump	Toboggan	Run	Walk	Bipedal	Quadrupedal
*Pygoscelis papua*	Gentoo Penguin	5–8.5	6.75	P	P	P	A	P	P	P
*Pygoscelis antarctica*	Chinstrap Penguin	3–5	4	P	P	P	A	P	P	P
*Pygoscelis adeliae*	Adélie Penguin	4–8	6	P	P	P	A	P	P	P
*Spheniscus mendiculus*	Galapagos Penguin	2–4	3	A	P	P	A	P	P	P
*Spheniscus humboldti*	Humboldt Penguin	4–6	5	A	P	A	A	P	P	A
*Spheniscus magellanicus*	Magellanic Penguin	3–6	4.5	A	P	A	A	P	P	A
*Spheniscus demersus*	African Penguin	2–3	2.5	A	P	P	A	P	P	P
*Eudyptula minor*	Little Penguin	1.5–1.5	1.5	A	P	A	A	P	P	A
*Megadyptes antipodes*	Yellow-Eyed Penguin	3–8.5	5.75	P	P	P	A	P	P	A
*Eudyptes chrysolophus*	Macaroni Penguin	5–6	5.5	P	P	A	A	P	P	A
*Eudyptes sclateri*	Erect-Crested Penguin	2.5–6	4.25	P	P	A	A	P	P	A
*Eudyptes robustus*	Snares Penguin	2.5–4	3.25	P	P	A	A	P	P	A
*Eudyptes pachyrhynchus*	Fiordland Penguin	2–5	3.5	P	P	A	A	P	P	A
*Eudyptes moseleyi*	Northern Rockhopper Penguin	2.4–4.3	3.35	P	P	A	P	P	P	A
*Eudyptes chrysocome*	Southern Rockhopper Penguin	2–3	2.5	P	P	A	P	P	P	A
*Aptenodytes forsteri*	Emperor Penguin	22–45	33.5	A	A	P	A	P	P	P
*Aptenodytes patagonicus*	King Penguin	9–18	13.5	A	P	P	A	P	P	P

Mass data were compiled from authoritative monographs and species accounts [48,49,50,51,52,53].

## Data Availability

No new data were created or analyzed in this study. Data sharing is not applicable to this article.

## Supplementary Materials

The following supporting information can be downloaded at: https://doi.org/10.5281/zenodo.20123368.
